# Fibroblast growth factor 10 ameliorates neurodegeneration in mouse and cellular models of Alzheimer's disease via reducing tau hyperphosphorylation and neuronal apoptosis

**DOI:** 10.1111/acel.13937

**Published:** 2023-07-28

**Authors:** Kaiming Guo, Wenting Huang, Kun Chen, Pengkai Huang, Wenshuo Peng, Ruiqing Shi, Tao He, Mulan Zhang, Hao Wang, Jian Hu, Xinshi Wang, Yangping Shentu, Huiqin Xu, Li Lin

**Affiliations:** ^1^ School of Pharmaceutical Sciences Wenzhou Medical University, University‐town Wenzhou China; ^2^ Oujiang Laboratory, Zhejiang Lab for Regenerative Medicine, Vision and Brain Health Wenzhou China; ^3^ The First Affiliated Hospital of Wenzhou Medical University Wenzhou China; ^4^ Jinhua Maternity and Child Health Care Hospital Jinhua China

**Keywords:** FGFR2/PI3K/AKT signaling pathway, fibroblast growth factor 10, neuronal apoptosis, tau hyperphosphorylation

## Abstract

Alzheimer's disease (AD) is characterized with senile plaques formed by Aβ deposition, and neurofibrillary tangles composed of hyperphosphorylated tau protein, which ultimately lead to cognitive impairment. Despite the heavy economic and life burdens faced by the patients with AD, effective treatments are still lacking. Previous studies have reported the neuroprotective effects of FGF10 in CNS diseases, but its role in AD remains unclear. In this study, we demonstrated that FGF10 levels were reduced in the serum of AD patients, as well as in the brains of 3xTg‐AD mice and APPswe‐transfected HT22 cells, suggesting a close relationship between FGF10 and AD. Further investigations revealed that intranasal delivery of FGF10 improved cognitive functions in 3xTg‐AD mice. Additionally, FGF10 treatment reduced tau hyperphosphorylation and neuronal apoptosis, thereby mitigating neuronal cell damage and synaptic deficits in the cortex and hippocampus of 3xTg‐AD mice, as well as APPswe‐transfected HT22 cells. Furthermore, we evaluated the therapeutic potential of FGF10 gene delivery for treating AD symptoms and pathologies. Tail vein delivery of the FGF10 gene using AAV9 improved cognitive and neuronal functions in 3xTg‐AD mice. Similarly, endogenous FGF10 overexpression ameliorated tau hyperphosphorylation and neuronal apoptosis in the cortex and hippocampus of 3xTg‐AD mice. Importantly, we confirmed that the FGFR2/PI3K/AKT signaling pathway was activated following intranasal FGF10 delivery and AAV9‐mediated FGF10 gene delivery in 3xTg‐AD mice and APPswe‐transfected HT22 cells. Knockdown of FGFR2 attenuated the protective effect of FGF10. Collectively, these findings suggest that intranasal delivery of FGF10 and AAV9‐mediated FGF10 gene delivery could be a promising disease‐modifying therapy for AD.

## Graphical abstract

Graphical representation depicting the potential mechanism involved in FGF10‐mediated protection against AD‐like symptoms and pathologies in both in vivo 3xTg‐AD mice and in vitro APPswe‐transfected HT22 cells. The neuroprotective effects of FGF10 were tested via intranasal delivery and AAV9‐mediated systemic gene delivery methods. FGF10 stimulates the FGFR2/PI3K/AKT signaling pathway, leading to the upregulation of p‐GSK3β S9/GSK3β and Bcl‐2/Bax. This, in turn, reduces tau hyperphosphorylation and neuronal apoptosis, resulting in protection against neuronal damage and cognitive dysfunction in AD.

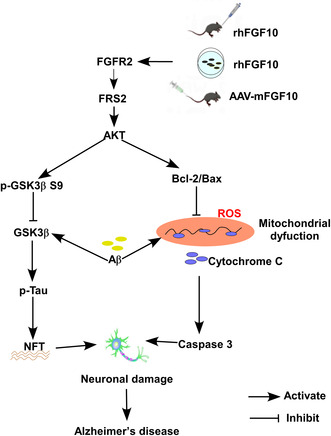

**Abbreviations**
AAV9: adeno‐associated virus serotype 9
AD: Alzheimer's disease
ALS: amyotrophic lateral sclerosis
Aβ: amyloid β‐peptide
BBB: blood‐brain barrier
CNS: central nervous system
DHE: dihydroethidium
ELISA: enzyme‐linked immunosorbent assay
FGF: fibroblast growth factor
GluR1: glutamate receptor R1
HD: Huntington's disease
HI: hypoxia/ischemia
LTP: long‐term potentiation
MACO: middle cerebral artery occlusion
MAP2: microtubule‐associated protein 2
MS: multiple sclerosis
MWM: Morris water maze
NFTs: neurofibrillary tangles
NOR: novel object recognition
OGD: oxygen/glucose deprivation
PD: Parkinson's disease
PSD95: postsynaptic density protein 95
ROS: reactive oxygen species
SCI: spinal cord injury
TBI: traumatic brain injury

## 1 INTRODUCTION

Alzheimer's disease (AD) is a devastating neurodegenerative disorder characterized by progressive neuronal loss, particularly in the cortex and hippocampus (Nussbaum & Ellis, 2003; Reisberg et al., 1987), resulting in memory impairment and cognitive decline (DeTure & Dickson, 2019). With aging being a significant risk factor, the incidence of AD is expected to rise as life expectancy increases, and it is projected that by 2050, the number of individuals affected by AD worldwide may exceed 100 million if effective prevention and treatment methods are not developed (Khachaturian, 1985; Mucke, 2009; Teipel et al., 2022). However, despite extensive research efforts, there is currently no accessible and effective disease‐modifying therapy for AD, underscoring the urgent need for novel therapeutic targets and strategies.

Neurofibrillary tangles (NFTs), composed of hyperphosphorylated tau, and senile plaques, formed by aberrant deposition of amyloid β‐peptide (Aβ), are two prominent pathological features of AD (Mattson, 2004). Aβ is known to be toxic to the central nervous system, leading to neuronal death. Furthermore, neurotoxicity induces hyperphosphorylation of tau protein, resulting in its aggregation and formation of NFTs. These NFTs disrupt axonal transport, compromise the structural stability of microtubules in neurons, and ultimately lead to neuronal cell death and cognitive decline (Cummings & Cole, 2002; Teipel et al., 2022). However, despite extensive research efforts, the cellular and molecular mechanisms underlying AD remain largely unknown, highlighting the urgent need for novel therapeutic targets and strategies.

The fibroblast growth factor (FGF) family, comprising a diverse group of growth factors, has been implicated in numerous biological and pathophysiological processes, such as angiogenesis, wound healing, embryonic development, and metabolism regulation (Ornitz & Itoh, 2001, 2015). Notably, emerging evidence suggests that FGFs also play a role in neurodegenerative diseases, including multiple sclerosis (MS) (Rajendran et al., 2021), Huntington's disease (HD) (La Spada, 2005), amyotrophic lateral sclerosis (ALS) (Cassina et al., 2005), Parkinson's disease (PD) (Liu et al., 2021) and AD (Alam et al., 2022). However, among the various FGFs, only FGF1, 2, 4, 9, 14, 21, and 23 have been extensively studied in the context of AD pathogenesis (Alam et al., 2022). The remaining FGFs have not yet been extensively investigated for their potential connection with AD due to a lack of research in this area. Further exploration is needed to elucidate the role of other FGFs in AD pathophysiology and to uncover their potential as therapeutic targets for AD treatment.

FGF10, a member of the FGF7/10/22 subfamily, is a paracrine protein that is expressed in various adult tissues, including the brain, vas deferens, lung, and white adipose tissue (Itoh & Ohta, 2014). FGF10 is reportedly involved in limb and lung formation (Sekine et al., 1999), lacrimal gland and mammary development (Mailleux et al., 2002; Makarenkova et al., 2000), otic placode induction, wound repair and tissue regeneration (Lv et al., 2019) and regulates thalamocortical axon guidance in the developing thalamus (Liu et al., 2020); its involvement in central nervous system (CNS) diseases is not well‐documented. Recent studies; however, have shed light on the potential neuroprotective effects of FGF10. FGF10 has been shown to exert a protective effect in models of oxygen/glucose deprivation (OGD) and middle cerebral artery occlusion (MACO), inhibiting neuroinflammation and apoptosis (Li et al., 2015, 2016). Neuron‐ and microglia/macrophage‐derived FGF10 has also been found to inhibit neuroinflammation and apoptosis in a spinal cord injury (SCI) model, and has been reported to have neuroprotective activity on the neurovascular unit in neonatal rat hypoxia/ischemia (HI) and traumatic brain injury (TBI) models (Chen et al., 2017; Fang et al., 2020; Hou et al., 2020). These findings suggest that FGF10 may possess neuroprotective effects in other CNS diseases as well. However, the specific effects of FGF10 and the underlying mechanisms involved in cognitive and memory function in AD remain largely unknown. Thus, the aim of our study was to investigate the potential neuroprotective benefits of FGF10 in AD using both in vivo and in vitro approaches, and elucidate the mechanisms by which FGF10 may suppress tau hyperphosphorylation and prevent neuronal apoptosis. Furthermore, we sought to explore the signaling pathways that mediate the positive effects of FGF10 and the processes underlying tau hyperphosphorylation and neuronal apoptosis in AD. Our findings suggest that FGF10 may represent a promising therapeutic approach for AD and other CNS diseases, warranting further investigation.

## 2 RESULTS

### 2.1 FGF10 levels are reduced in the serum of AD patients as well as in mouse and cellular AD models

**FIGURE 1:**
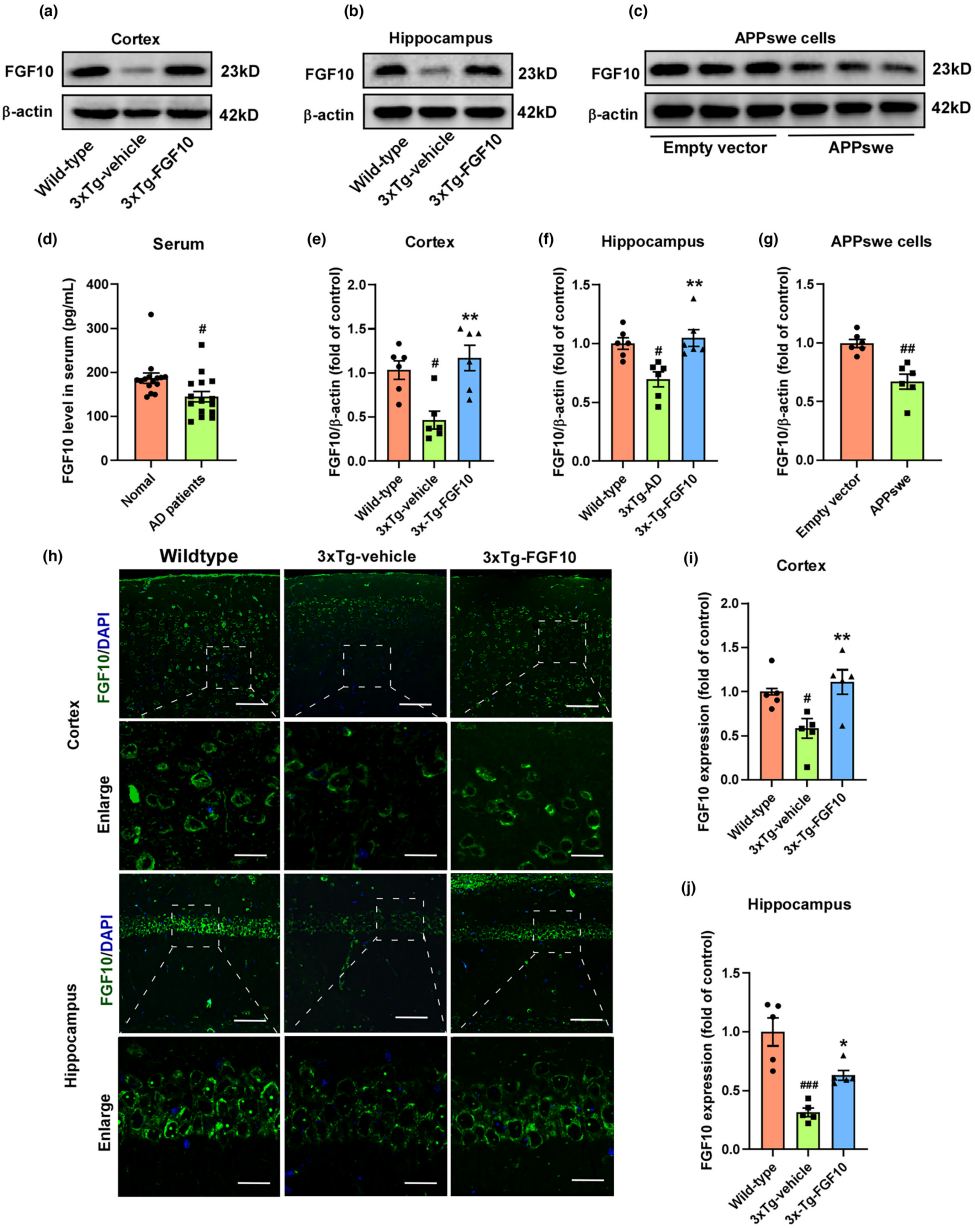
FGF10 deficiency in serum of AD patients as well as mouse and cellular AD models. (a,b) The levels of FGF10 in the cortex and hippocampus of mice were measured by western blotting. (c) The levels of FGF10 in APPswe‐transfected HT22 cells were measured by western blotting. (d) The levels of FGF10 in serum of AD patients were examined by ELISA test (*n* = 15 samples per group). (e,f) Densitometric analyses were performed on the immunoreactivities observed in panels a,b (*n* = 5 mice per group). (g) Densitometric analyses were performed on the immunoreactivities observed in panels c (*n* = 6 replicates per group). (h) The expression of FGF10 in the cortex and hippocampus was evaluated by immunofluorescence staining. Upper column: scale bar, 100 μm; Lower column: scale bar, 25 μm. (i,j) Quantification of immunofluorescence staining in h (*n* = 5 mice per group). In the ELISA test, #*p* < 0.05 compared with the normal group. Data are presented as the mean values ± SEM. In in vivo experiments, #*p* < 0.05 and ###*p* < 0.001 compared with the wild‐type mice; *p < 0.05 and **p < 0.01 compared with the 3xTg‐vehicle mice. In in vitro experiments, #*p* < 0.05 compared with the empty vector group. Data are presented as. the mean values ± SEM.

To determine whether FGF10 levels are dysregulated in AD, we first measured FGF10 levels in the serum of AD patients using enzyme‐linked immunosorbent assay (ELISA). Our results showed a significant decrease in FGF10 levels in AD patients compared to age‐matched controls (Figure 1d). To further validate these findings, we examined FGF10 levels in mouse models of AD. Cortices and hippocampi were collected from 11‐month‐old female 3xTg‐AD and wild‐type mice, which are known to exhibit cognitive decline and prominent Aβ deposits and pathological tau. FGF10 levels were assessed using western blotting (Figure 1a,b) and immunofluorescence staining (Figure 1h). Our western blotting results revealed a significant reduction in FGF10 levels in the cortex and hippocampus of 3xTg‐AD mice compared to wild‐type mice, with an increase in FGF10 levels observed after intranasal administration of FGF10 (Figure 1e,f). Immunofluorescence staining also demonstrated a decrease in FGF10 expression intensity in the cortex and hippocampus of 3xTg‐AD mice (Figure 1i,j). Additionally, we observed a significant increase in FGF10 levels in the cortex and hippocampus after intranasal administration of FGF10, indicating successful delivery of FGF10 to these brain regions. Furthermore, we investigated FGF10 expression in HT22 cells transfected with APPswe, which expresses the “Swedish” variant of amyloid precursor protein. Our results revealed a significant difference in FGF10 expression between empty vector‐transfected cells and APPswe‐transfected cells, with the latter showing significantly lower FGF10 expression (Figure 1g).

### 2.2 FGF10 improves cognitive functions in 3xTg‐AD mice

**FIGURE 2:**
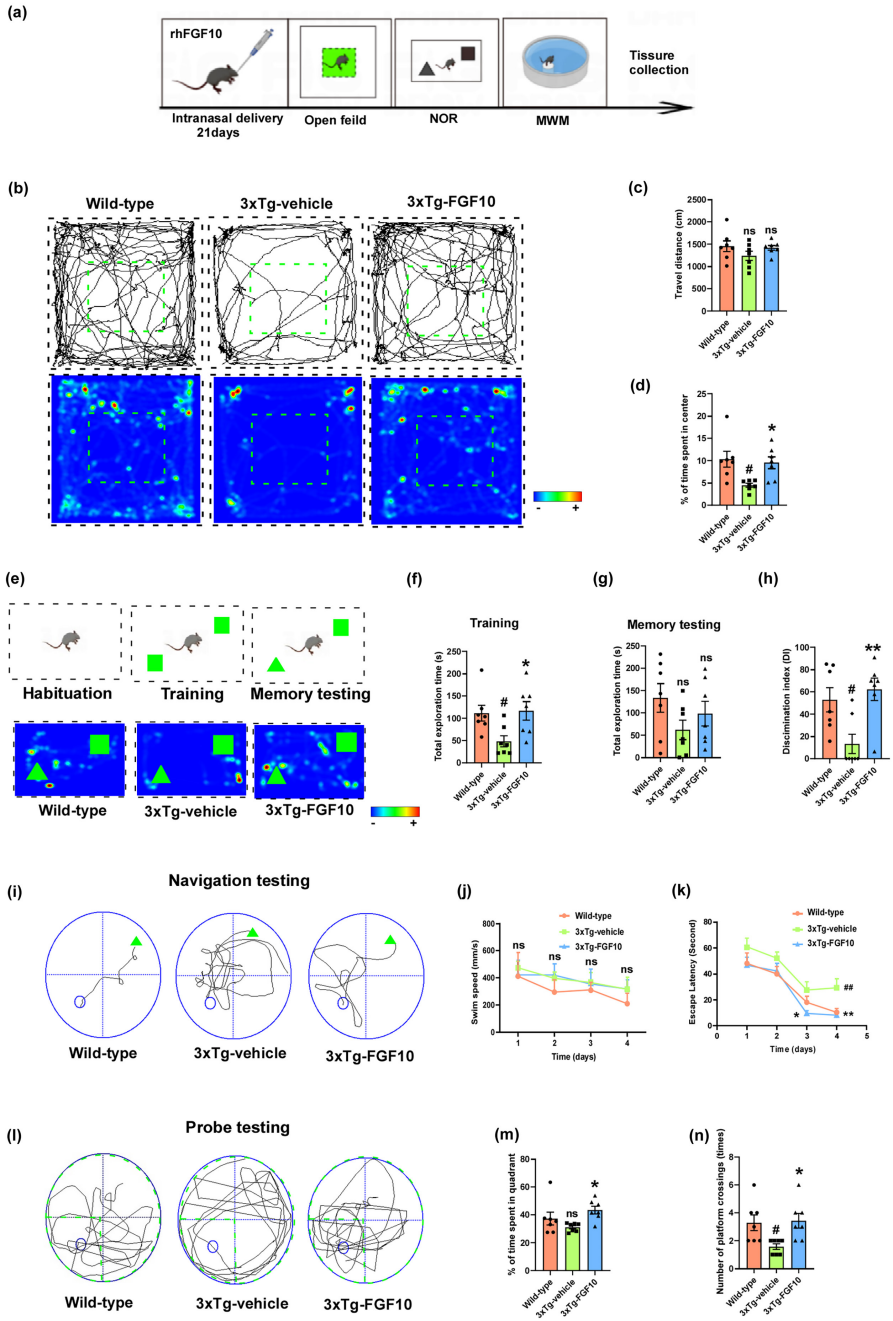
The beneficial effect of FGF10 on cognitive performance in 3xTg‐AD mice. (a) Experimental design. (b) Open Field Test for assessing anxiety and depression behavior. Representative path tracking map (upper column) and path tracking heat map (lower column), total travel distance (c), and % of time spent in the center (d) were recorded and analyzed. (e) NOR test to evaluate cognitive performance in different groups of mice. Diagram of the NOR test (upper column) and representative path tracking heat map (lower column) were shown. Total exploration time towards objects in the training (f) and memory testing (g) phases, and discrimination index (h) were recorded and analyzed. MWM for testing spatial cognitive performance. Representative swimming paths (i), average swimming speed (j), and escape latency (k) in the navigation testing phase; and representative swim paths (l), % time spent in the target quadrant (m), and number of platform crossings (n) in the probe testing phase were shown. #*p* < 0.05 and ##*p* < 0.01 compared with the wild‐type mice; *p < 0.05 and **p < 0.01 compared with the 3xTg‐vehicle mice. Data are presented as the mean ± SEM (*n* = 7 mice per group).

AD patients often exhibit anxiety‐ and depression‐like behaviors, as reported in previous studies (Mendez, 2021; Teri et al., 1999). In this study, we examined the effects of intranasal delivery of vehicle or FGF10 for 21 days on anxiety‐ and depression‐like behaviors in mice using the open field test. We found no significant differences in total travel distance among the three groups of mice (Figure 2c). However, 3xTg‐vehicle mice spent less time in the center of the open field compared to wild‐type mice, while FGF10 treatment increased the time spent in the center, suggesting that FGF10 may alleviate anxiety‐ and depression‐like behaviors in 3xTg‐AD mice (Figure 2d). Before conducting the novel object recognition (NOR) test and Morris water maze (MWM) test, the mice underwent evaluations including the rotarod test, visual cliff test, and visible platform test in the Morris water maze. These evaluations aimed to assess and eliminate potential disparities in visual acuity, motor ability, and motivation between the 11‐month‐old 3xTg mice and wild‐type mice. Remarkably, the results of these experiments revealed no significant differences in motor, visual, and motivation function between the two groups of mice (Figure S1). During the training phase of the NOR test, mice were exposed to two identical objects. After 3 h of the training session, the mice were exposed to a familiar object and a new object in the testing session (Figure 2e). We found that the total exploration time of the two objects in 3xTg‐vehicle mice was less than that in wild‐type mice in both the training and test phases, while FGF10 treatment increased the total exploration time for the two objects. These findings indicate that 3xTg‐vehicle mice had reduced exploratory behavior, which was restored by FGF10 treatment (Figure 2f,g). Furthermore, compared to wild‐type mice, 3xTg‐vehicle mice showed impaired ability to recognize the novel object; whereas, FGF10 treatment restored this ability (Figure 2h). We also assessed spatial cognitive function using the MWM test. We found that the average escape latency in searching for the target platform decreased with training. However, 3xTg‐vehicle mice exhibited a longer latency, indicating a significant decline in spatial learning and memory compared to wild‐type mice. This increased escape latency was attenuated by FGF10 treatment (Figure 2k). Moreover, swimming speed did not significantly differ among the groups during the training period, indicating no motor disturbance in the treated animals (Figure 2j). Following 4 days of training, a spatial probe test was conducted on the fifth day (Figure 2l). The number of platform crossings and the time spent in the target quadrant were lower in 3xTg‐vehicle mice compared to wild‐type mice, but FGF10 treatment increased the number of crossings and the swimming time in the target quadrant (Figure 2m,n). These results collectively suggest that FGF10 improves cognitive function in 3xTg‐AD mice.

### 2.3 FGF10 ameliorates neuronal damage and synaptic deficits in 3xTg‐AD mice and APPswe‐transfected HT22 cells

**FIGURE. 3:**
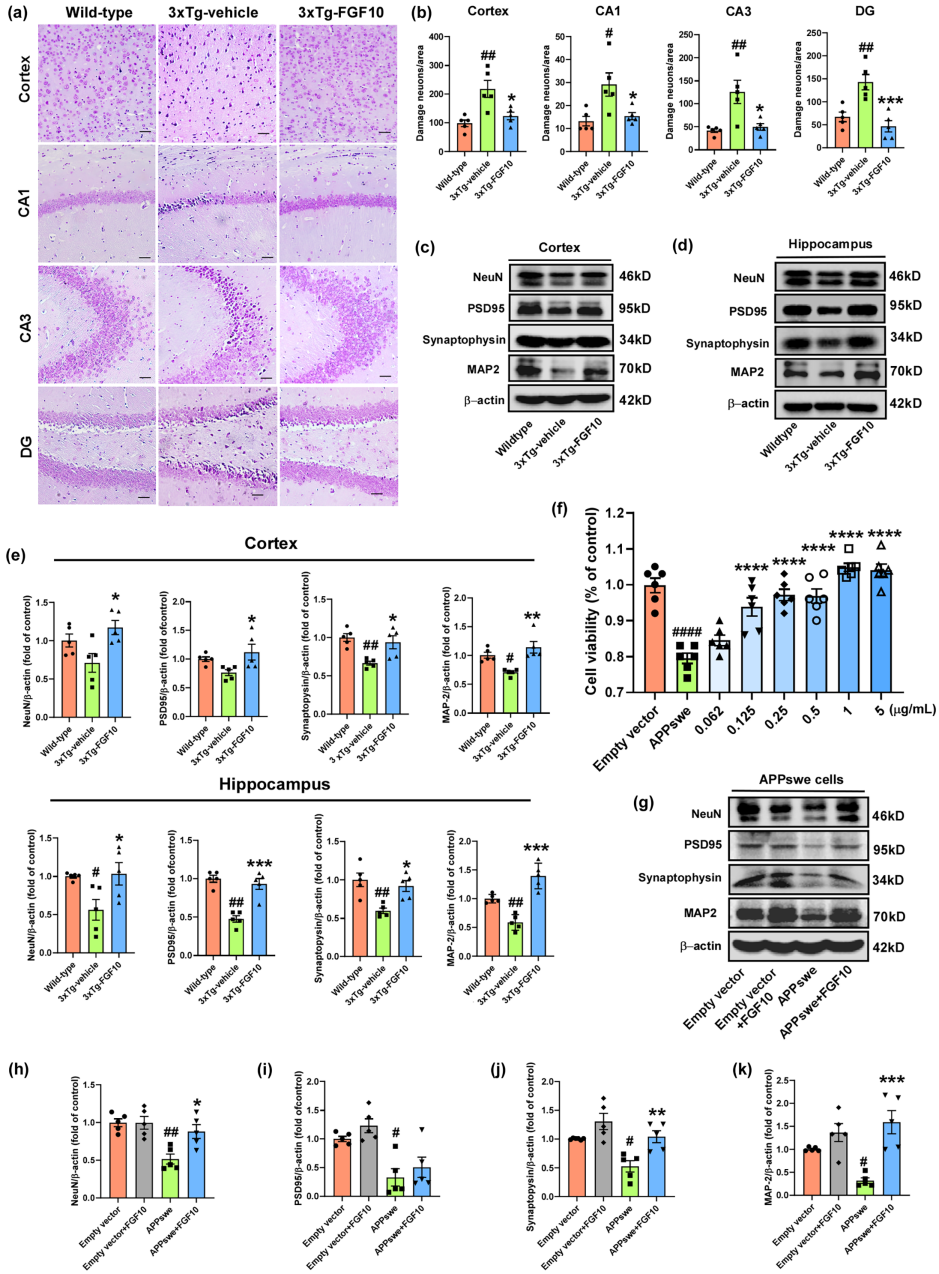
The effects of FGF10 on neuronal damage and synaptic deficits in vivo and in vitro. (a) Nissl staining was performed to assess neuronal function in the cortex and hippocampus (CA1, CA3, and DG) of wild‐type and 3xTg‐AD mice. Scale bar, 25 μm. (b) Statistical analysis results of Nissl staining (*n* = 5 mice per group). (c,d) The levels of NeuN, PSD95, synaptophysin, MAP2, and β‐Actin in the cortex and hippocampus of wild‐type and 3xTg‐AD mice were detected by western blotting. (e) Densitometric analyses were performed on the immunoreactivities observed in panels c and d (*n* = 5 mice per group). (f) Neuroprotective effect of FGF10 in APPswe‐transfected HT22 cell viability (*n* = 6 replicates per group). (g) The levels of NeuN, PSD95, synaptophysin, MAP2 and β‐Actin in APPswe‐transfected HT22 cells were detected by western blotting (*n* = 5 replicates per group). (h–k) Densitometric analyses were performed on the immunoreactivities observed in panel g. In in vivo experiments, #*p* < 0.05 and ##*p* < 0.01 compared with the wild‐type mice; *p < 0.05, **p < 0.01, and ***p < 0.001 compared with the 3xTg‐vehicle mice. In in vitro experiments, #*p* < 0.05, ##*p* < 0.01, and ####*p* < 0.0001 compared with the empty vector cells; *p < 0.05, **p < 0.01, ***p < 0.001, and ****p < 0.0001 compared with the APPswe cells. Data are presented as mean ± SEM.

To elucidate the underlying mechanisms responsible for the cognitive improvement observed in 3xTg‐AD mice, we conducted Nissl staining on harvested tissues. Nissl staining revealed morphological changes in neurons in the cortex, CA1, CA3, and DG regions of the hippocampus (Figure 3a). Compared to wild‐type mice, 3xTg‐vehicle mice exhibited numerous damaged neurons in these regions, characterized by shrunken cytoplasm and condensed staining. In contrast, these degenerative changes were alleviated in 3xTg‐FGF10 mice (Figure 3b). Neuronal loss and synaptic deficits are known to be associated with cognitive decline in AD. Therefore, we further examined whether treatment with FGF10 could ameliorate neuronal markers of neuron and synaptic plasticity in the cortex and hippocampus of 3xTg‐AD mice. We found that the levels of NeuN, postsynaptic density protein 95 (PSD95), synaptophysin, and microtubule‐associated protein 2 (MAP2) were significantly lower in 3xTg‐vehicle mice compared to wild‐type mice, but these deficits were mitigated in 3xTg‐FGF10 mice (Figure 3c–e). Moreover, we conducted western blotting experiments to assess the total and phosphorylation levels of glutamate receptor R1 (GluR1) and CaMKII, two crucial proteins involved in the long‐term potentiation (LTP) pathway. Specifically, we observed a significant decrease in the ratio of phosphorylated and total GluR1 and CaMKII in 3xTg‐AD mice, which is consistent with previous reports. Remarkably, our results demonstrated that FGF10 treatment effectively upregulated the phosphorylation to total ratio of both GluR1 and CaMKII in the cortex and hippocampus of 3xTg‐AD mice (Figure S3). Lastly, we investigated whether FGF10 could ameliorate neuronal damage and synaptic deficits in APPswe‐transfected HT22 cells, an in vitro AD model. We performed CCK8 assays to assess cell viability in empty vector‐ and APPswe‐transfected HT22 cells with or without FGF10. Quantitative analysis revealed that the APPswe group had reduced cell viability compared to the empty vector group, but FGF10 intervention increased cell viability, particularly at high concentrations (1 and 5 μg/mL) (Figure 3f). We also evaluated markers of neuronal and synaptic plasticity in APPswe‐transfected HT22 cells using western blotting (Figure 3g). Our results showed that APPswe cells exhibited decreased levels of PSD95, synaptophysin, and MAP2, which were reversed by FGF10 intervention (Figure 3h–k). Collectively, based on the findings from the in vivo and in vitro AD models, we confirmed that FGF10 has the potential to mitigate neuronal damage and synaptic deficits in 3xTg‐AD mice and APPswe‐transfected HT22 cells, suggesting the potential of FGF10 as a promising intervention for restoring synaptic dysfunction in the context of AD.

### 2.4 FGF10 reduces tau hyperphosphorylation in 3xTg‐AD mice and APPswe‐transfected HT22 cells

**FIGURE. 4:**
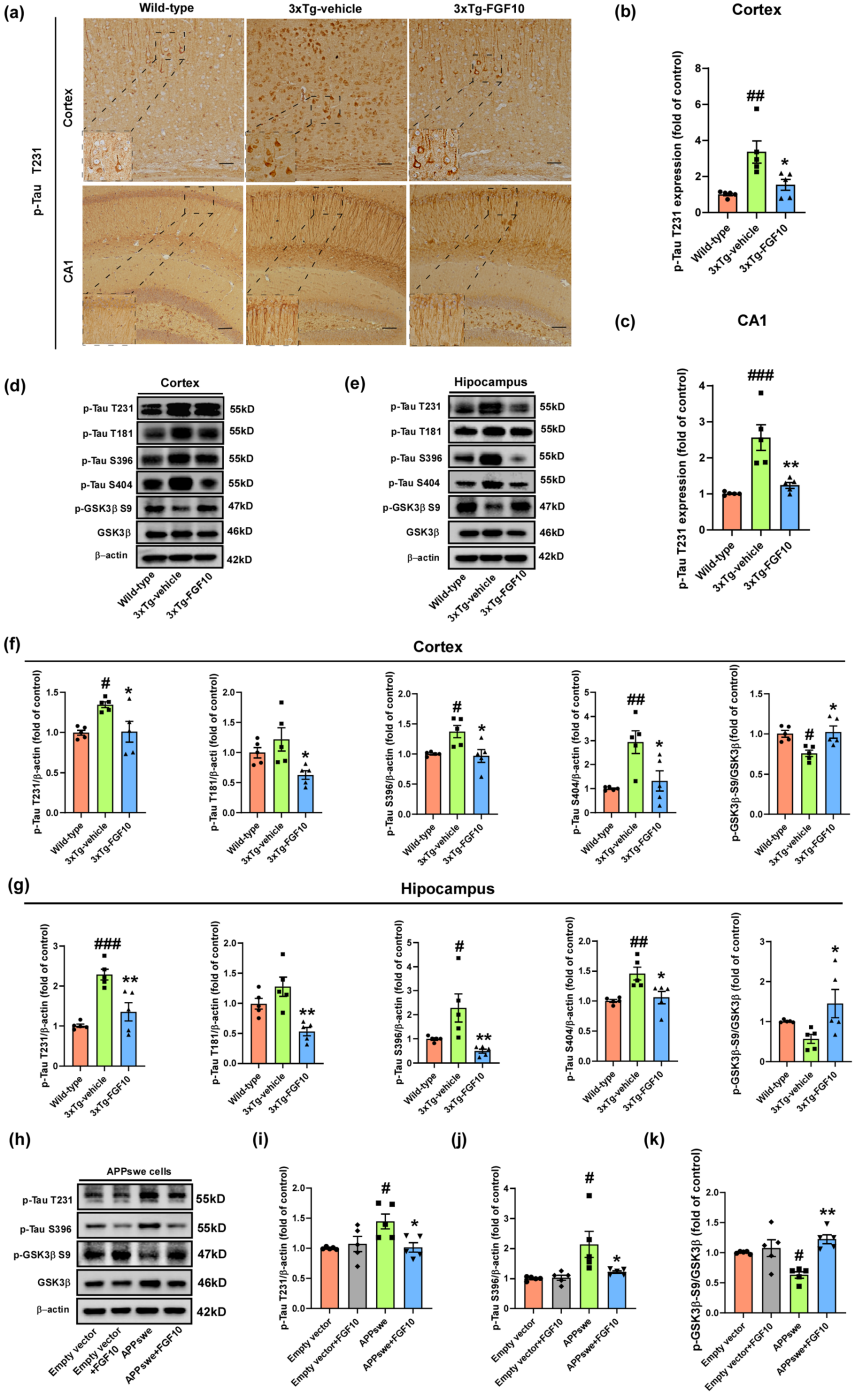
The effect of FGF10 against tau hyperphosphorylation in vivo and in vitro. (a) Immunohistochemistry images of p‐Tau T231 in the cortex and hippocampus of wild‐type and 3xTg‐AD mice, with enlarged images on the left bottom. Scale bar, 25 μm. (b,c) Statistical analysis results of immunohistochemistry of p‐Tau T231 (*n* = 5 mice per group). (d,e) Levels of p‐Tau T231, p‐Tau T181, p‐Tau S396, p‐Tau S404, GSK3b, and p‐GSK3b S9 in the cortex and hippocampus of wild‐type and 3xTg‐AD mice were detected by western blotting. (f,g) Densitometric analyses were performed on the immunoreactivities observed in panels d and e (*n* = 5 mice per group). (h) Levels of p‐Tau T231, p‐Tau S396, GSK3β, and p‐GSK3β S9 in APPswe‐transfected HT22 cells were detected by western blotting. (i–k) Densitometric analyses were performed on the immunoreactivities observed in panel h. (*n* = 5 replicates per group). In in vivo experiments, #*p* < 0.05, ##*p* < 0.01, and ###*p* < 0.001 compared with the wild‐type mice; *p < 0.05 and **p < 0.01 compared with the 3xTg‐vehicle mice. In in vitro experiments, #*p* < 0.05, ##*p* < 0.01, and ###*p* < 0.001 compared with the empty vector cells; *p < 0.05 and **p < 0.01 compared with the APPswe cells. Data are presented as mean ± SEM.

The presence of NFTs of hyperphosphorylated tau protein is one of the main characteristic pathologies in AD brain that contributes to cognitive disorders in patients with AD (Bloom, 2014). In order to investigate the impact of FGF10 on tau hyperphosphorylation, we performed immunohistochemical staining against phosphorylated tau at threonine 231 (T231) in the cortex and CA1 region of the hippocampus (Figure 4a). Our results revealed significantly higher T231 expression in the cortex and CA1 region of 3xTg‐vehicle mice compared to wild‐type mice, indicating an increase in tau hyperphosphorylation in the 3xTg‐AD mice. However, treatment with FGF10 effectively reversed these elevations (Figure 4b,c). Furthermore, we conducted western blot analysis to assess tau phosphorylation at serines 404 and 396 (S404 and S396, respectively), T231, and threonine 181 (T181), as well as the ratio of phosphorylated GSK3β at serine 9 (p‐GSK3β S9) to total GSK3β in the cortex and hippocampus (Figure 4d,e). Notably, FGF10 treatment significantly reduced the levels of tau hyperphosphorylation at S404, S396, T231, and T181, and increased the p‐GSK3β S9/GSK3β ratio compared to 3xTg‐vehicle mice (Figure 4f,g). In addition, we also investigated the effect of FGF10 on tau phosphorylation in vitro using APPswe cells (Figure 4h). Our findings revealed increased levels of tau phosphorylation at T231 and S396, as well as a reduced p‐GSK3β S9/GSK3β ratio in APPswe cells. However, treatment with FGF10 significantly attenuated tau phosphorylation at S396 and increased the p‐GSK3β S9/GSK3β ratio in APPswe cells (Figure 4i–k). Taken together, these results suggest that FGF10 treatment effectively reduces tau hyperphosphorylation both in vivo and in vitro, indicating its potential therapeutic benefits in ameliorating AD pathologies.

### 2.5 FGF10 ameliorates neuronal apoptosis in 3xTg‐AD mice and APPswe‐transfected HT22 cells

**FIGURE. 5:**
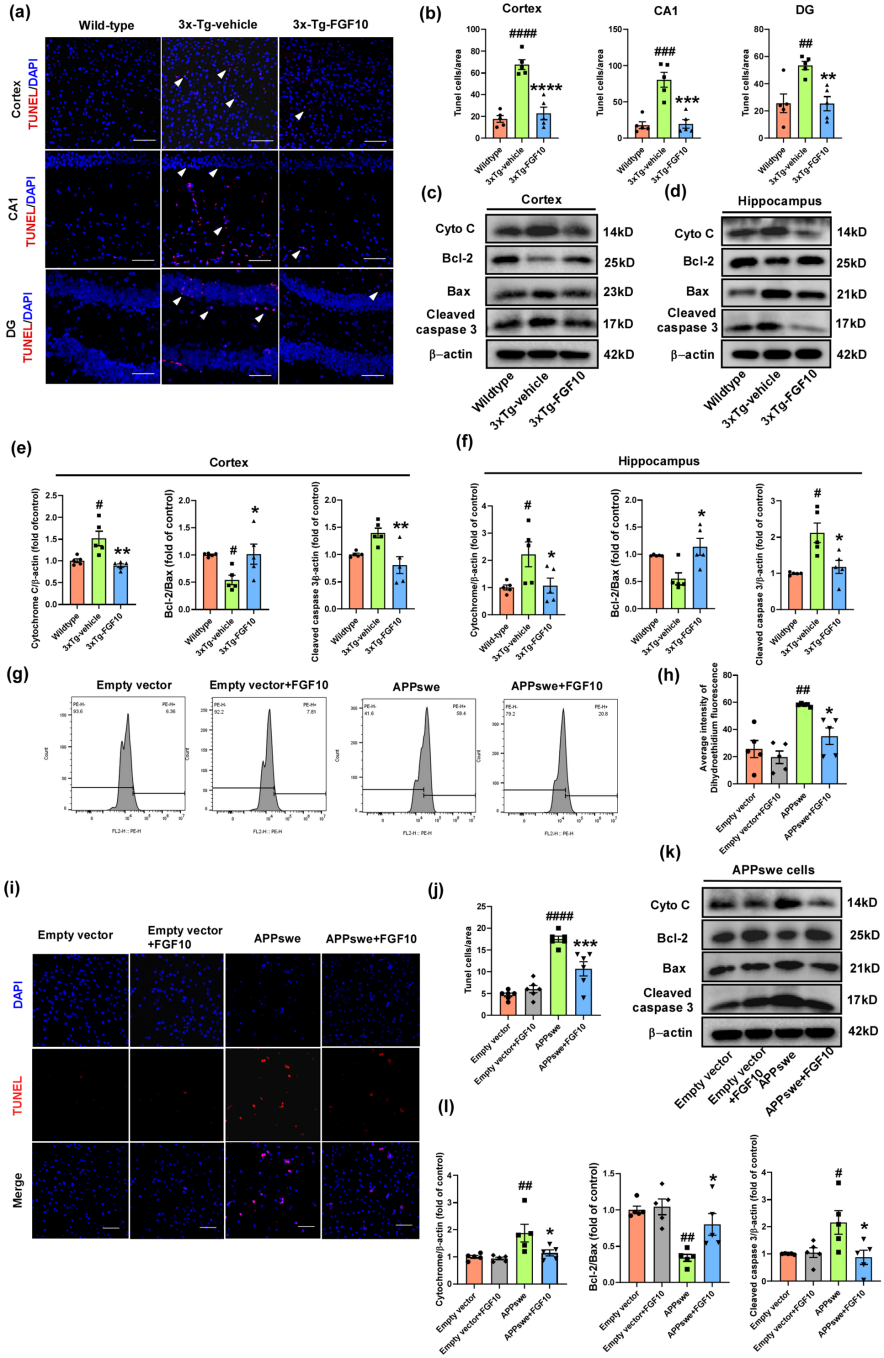
The effect of FGF10 against neuronal apoptosis in vivo and in vitro. (a) The effect of FGF10 against neuronal apoptosis in the cortex and hippocampus (CA1 and DG) of wildtype and 3xTg‐AD mice was evaluated by TUNEL assay, with white arrows indicating TUNEL‐positive cells (scale bar, 100 μm). (b) Quantification of TUNEL staining in a (*n* = 5 mice per group). (c,d) Levels of cytochrome c (abbreviated as Cyt C), Bcl‐2, Bax, and cleaved caspase 3 (active form of caspase 3) along with β‐actin in the cortex and hippocampus were detected by western blotting. (e,f) Densitometric analyses were performed on the immunoreactivities observed in panels c and d (*n* = 5 mice per group). (g) Reactive oxygen species (ROS) detection by flow cytometry after DHE staining. (h) Average intensity of DHE fluorescence in G. (*n* = 5 replicates per group). (i) The effect of FGF10 against neuronal apoptosis in APPswe‐transfected HT22 cells was evaluated by TUNEL assay (scale bar, 100 μm). (j) Quantitation of TUNEL staining in I (*n* = 5 replicates per group). (k) Levels of cytochrome c (abbreviated as Cyt C), Bcl‐2, Bax, and cleaved caspase 3 (active form of caspase 3) along with β‐actin in APPswe‐transfected HT22 cells were detected by western blotting. (l) Quantification of representative protein bands from western blotting in k (*n* = 5 replicates per group). In in vivo experiments, #*p* < 0.05, ##*p* < 0.01, ###*p* < 0.001, and ####*p* < 0.0001 compared with the wild‐type mice; *p < 0.05, **p < 0.01, ***p < 0.001, and ****p < 0.0001 compared with the 3xTg‐vehicle mice. In in vitro experiments, #*p* < 0.05, ##*p* < 0.01, and ####*p* < 0.0001 compared with the empty vector cells; *p < 0.05 and ***p < 0.001 compared with the APPswe cells. Data are presented as mean ± SEM.

Neuronal apoptosis, a hallmark feature of AD associated with memory impairment, was investigated in vivo and in vitro to assess the effects of FGF10. TUNEL staining and western blotting were utilized for analysis. Compared to wild‐type mice, 3xTg‐vehicle mice exhibited prominent apoptosis; whereas, 3xTg‐FGF10 mice showed a significant reduction in TUNEL‐positive cells in the cortex and hippocampal regions, including CA1, CA3, and DG (Figure 5a,b). Western blotting revealed that in 3xTg‐vehicle mice, the expression of proapoptotic factors, including cytochrome c, Bax, and cleaved caspase‐3, was upregulated, while the expression of Bcl‐2, an apoptosis inhibitor, was downregulated compared to wild‐type mice. However, FGF10 treatment reversed these effects (Figure 5c–f). In cellular experiments, we hypothesized that FGF10 may modulate intracellular reactive oxygen species (ROS) levels in HT22 cells transfected with APPswe, which were assessed using the fluorescent probe dihydroethidium (DHE). APPswe cells exhibited significantly elevated ROS levels, which were reduced upon FGF10 treatment, indicating a potential antioxidative effect of FGF10 in APPswe‐transfected HT22 cells (Figure 5g,h). TUNEL staining revealed that APPswe cells had increased TUNEL‐positive cells compared to empty vector cells, but FGF10 treatment decreased the number of TUNEL‐positive cells (Figure 5i,j). Western blotting analysis showed that FGF10 treatment did not affect the expression of apoptosis regulators in empty vector cells, but in APPswe cells, the expression of cytochrome c, Bax, and cleaved caspase‐3 was increased, while Bcl‐2 expression was decreased. Remarkably, FGF10 intervention attenuated the expression of cytochrome c, Bax, and cleaved caspase 3, and increased the expression of Bcl‐2 in APPswe cells (Figure 5k,l). In conclusion, these results demonstrate that FGF10 ameliorates neuronal apoptosis in 3xTg‐AD mice and APPswe‐transfected HT22 cells.

### 2.6 Endogenous FGF10 overexpression improves cognitive and synaptic deficits in 3xTg‐AD mice

**FIGURE. 6:**
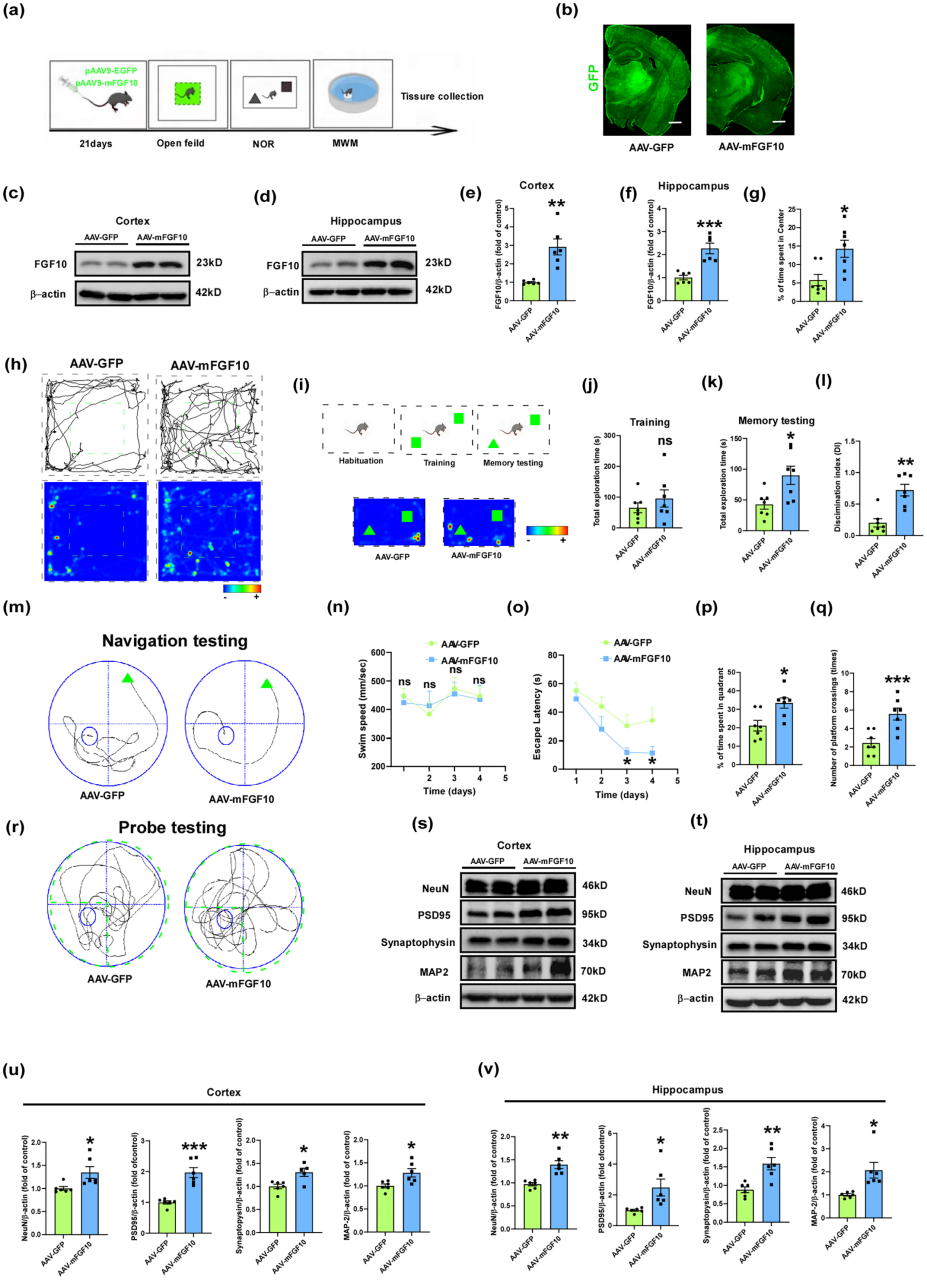
Endogenous FGF10 overexpression improves cognitive and synaptic deficits in 3xTg‐AD mice. (a) Experimental design. (b) Representative images of coronal brain sections from 21 days post‐injection with AAV‐GFP and AAV‐mFGF10 in 3xTg‐AD mice (scale bar, 500 μm). (c,d) Levels of FGF10 and β‐Actin in cortex and hippocampus of 3xTg‐AD mice were determined by western blotting. (e,f) Densitometric analyses were performed on the immunoreactivities observed in panels c and d (*n* = 6 mice per group). (h) The Open Field Test was performed to assess anxiety and depression‐like behavior in 3xTg‐AD mice. Representative path tracking maps (upper column) and mouse path tracking heat maps (lower column), as well as percentage of time spent in the center (g) were recorded and analyzed. (i) NOR test was conducted to evaluate cognitive performance, showing the diagram of the NOR test (upper column) and representative path tracking heat map (lower column). Total exploration time towards objects during the training (j) and memory testing (k) phases, as well as discrimination index (l), were recorded and analyzed (*n* = 7 mice per group). MWM was performed to test spatial cognitive performance, showing the representative swim paths during the navigation testing phase (m), average swimming speed (n), and escape latency (o) in training trials, as well as the representative swim paths during the probe testing phase (r), percentage of time spent in the target quadrant (p) and number of platform crossings (q) in the spatial probe test (*n* = 7 mice per group). (s,t) Levels of NeuN, PSD95, synaptophysin, MAP2, and β‐Actin in cortex and hippocampus of 3xTg‐AD mice were determined by western blotting. (u,v) Densitometric analyses were performed on the immunoreactivities observed in panels s and t (*n* = 6 mice per group). *p < 0.05, **p < 0.01, and ***p < 0.01 compared with the AAV‐GFP mice. Data are presented as mean values ± SEM.

It has been reported that adeno‐associated virus (AAV) serotype 9 (AAV9) virus can pass the blood–brain barrier (BBB) and achieve a good infection effect in the brain. AAV9 vectors are safe and convenient with low toxicity and are considered to be ideal CNS disease treatments (Fischell & Fishman, 2021; Saraiva et al., 2016). We conducted further investigations to assess the protective effect of endogenous FGF10 on AD and explored different methods of administration and treatment. We administered an AAV carrying mFGF10 overexpression through tail vein injection. After 21 days, we performed behavioral tests including open field, NOR, and MWM tests. Once the behavioral experiments were completed, the mice were sacrificed and brain tissues were collected for subsequent experiments (Figure 6a). To determine the brain penetration efficiency and neural tissue transduction of the AAV, we performed fluorescence imaging and western blot analysis on brain sections. GFP was used to visualize viral transduction, and both AAV‐GFP and AAV‐mFGF10 showed high intensity throughout the brain (Figure 6b). Western blotting revealed elevated levels of FGF10 expression in the cortex and hippocampus of AAV‐mFGF10 mice compared to AAV‐GFP mice (Figure 6c–f). We assessed anxiety‐ and depression‐like behavior in 3xTg‐AD mice using the open field test. AAV‐mFGF10 mice spent more time in the center of the open field compared to AAV‐GFP mice (Figure 6g,h). Cognitive performance was evaluated using the NOR test, and AAV‐mFGF10 mice spent more time exploring objects in both the training and test phases, with a higher discrimination index (DI), compared to AAV‐GFP mice (Figure 6i–l). Spatial memory was assessed using the MWM test, and AAV‐mFGF10 mice showed no difference in swimming speed but had a shorter escape latency compared to AAV‐GFP mice (Figure 6m–o). In the spatial probe test, AAV‐mFGF10 mice had more platform crossings and spent more time in the target quadrant compared to AAV‐GFP mice (Figure 6p–r). As cognitive decline is associated with synaptic deficits, we further evaluated alterations in synaptic proteins. Western blotting analysis showed that AAV‐mFGF10 mice had higher levels of neuronal and synaptic plasticity‐related proteins, including NeuN, PSD95, synaptophysin, and MAP2, in the cortex and hippocampus compared to AAV‐GFP mice (Figure 6s–v). In addition, we investigated the proteins associated with LTP and found that AAV‐mFGF10 treatment significantly upregulated the phosphorylation to total protein ratio of both GluR1 and CaMKII in the cortex and hippocampus of 3xTg‐AD mice (Figure S4). This observation suggests that endogenous FGF10 overexpression can upregulate LTP related molecules expression, thereby improving synaptic deficits in 3xTg‐AD mice. The upregulation of the phosphorylation to total protein ratio of GluR1 and CaMKII indicates that FGF10 treatment has the ability to modulate synaptic plasticity mechanisms, ultimately leading to improved synaptic deficits. These findings collectively demonstrate that endogenous overexpression of FGF10 improves cognitive and synaptic deficits in 3xTg‐AD mice, indicating the beneficial effects of AAV‐mFGF10 treatment on cognitive and synaptic deficits in AD.

### 2.7 Endogenous FGF10 overexpression ameliorates tau hyperphosphorylation and neuronal apoptosis in 3xTg‐AD mice

To elucidate the impact of AAV‐mFGF10 on tau hyperphosphorylation and neuronal apoptosis in the 3xTg‐AD mouse model, we conducted a comprehensive analysis of p‐Tau 231 levels in the cortex and hippocampus using immunofluorescence staining. Strikingly, our findings, as illustrated in Figure S1a,b, revealed that AAV‐mFGF10 mice exhibited a substantial reduction in p‐Tau T231 levels in both the cortex and hippocampus compared to AAV‐GFP mice. Following this, we employed western blotting to examine the levels of tau phosphorylation at multiple sites, including T231, T181, S404, and S396, as well as the ratio of p‐GSK3β S9 to GSK3β, in the cortex and hippocampus. Remarkably, AAV‐mFGF10 mice exhibited significantly lower levels of phosphorylation at T231, T181, S404, and S396 compared to AAV‐GFP mice, accompanied by an enhanced ratio of p‐GSK3β S9 to GSK3β (Figure S1c–e), indicative of a reduction in tau hyperphosphorylation. To further investigate the potential anti‐apoptotic effects of endogenous FGF10 overexpression, we performed TUNEL assay to evaluate apoptosis in the cortex and CA1 and DG regions of the hippocampus. Notably, AAV‐GFP mice exhibited a marked increase in apoptosis, while AAV‐mFGF10 mice displayed a robust protection against AD‐induced apoptosis in these areas (Figure S1f,g), suggesting a mitigating effect of AAV‐mFGF10 on neuronal apoptosis in 3xTg‐AD mice. To substantiate these findings, we examined apoptosis‐related proteins using western blot analysis. Consistently, the expression of cytochrome c and cleaved caspase‐3, key markers of apoptosis, was elevated in AAV‐GFP mice but attenuated in AAV‐mFGF10 mice, both in the hippocampus and cortex. Furthermore, the anti‐apoptotic protein Bcl‐2 exhibited higher expression in AAV‐mFGF10 mice compared to AAV‐GFP mice, whereas the pro‐apoptotic protein Bax displayed an opposite trend (Figure S1h–k). Collectively, our results provide compelling evidence that endogenous FGF10 overexpression ameliorates tau hyperphosphorylation and neuronal apoptosis in 3xTg‐AD mice.

### 2.8 The FGFR2/PI3K/AKT signaling pathway may be involved in the neuroprotective effect of FGF10

**FIGURE. 7:**
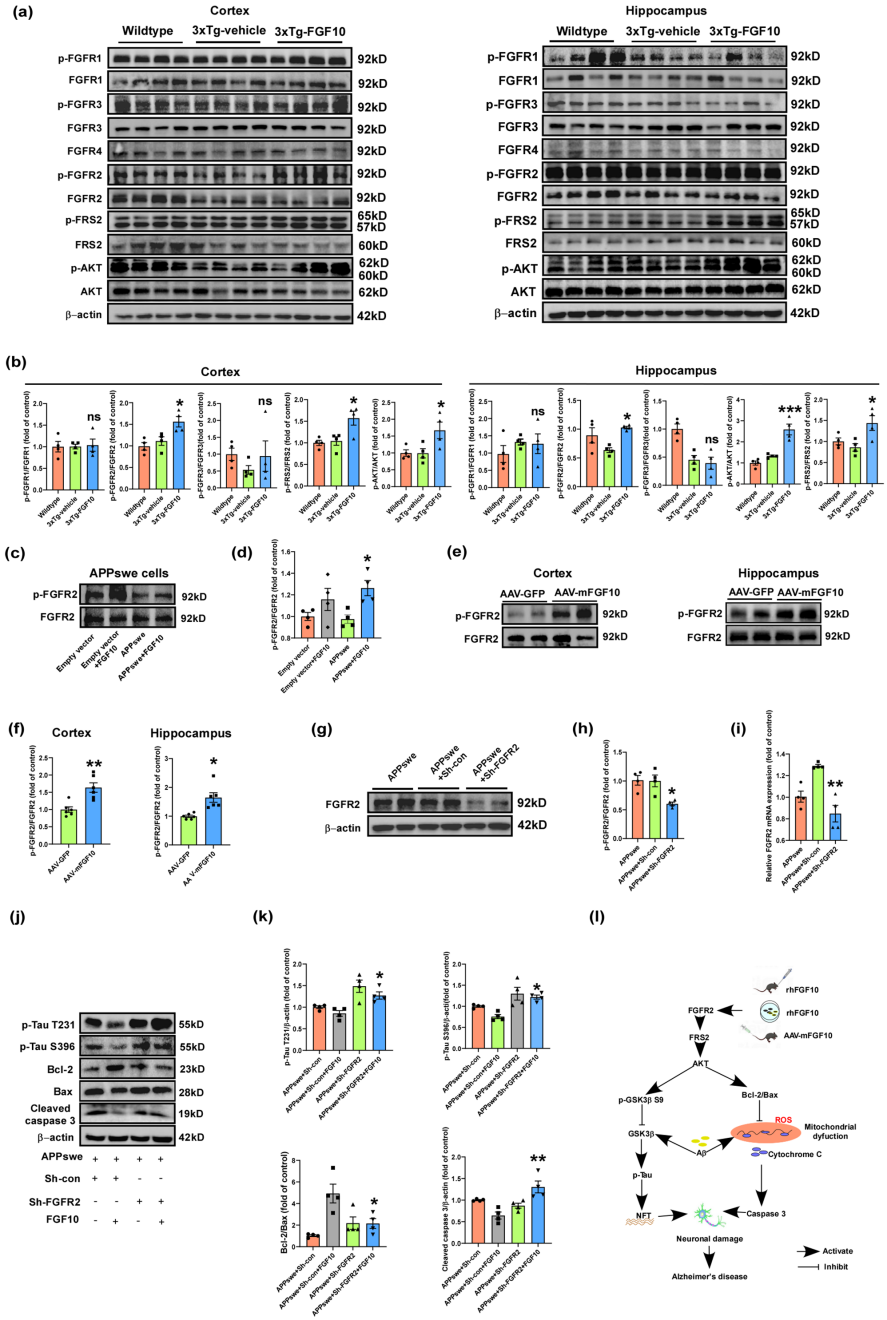
The role of FGFR2/PI3K/AKT pathway in the neuroprotective effect of FGF10 against tau hyperphosphorylation and neuronal apoptosis. (a) The phosphorylation and total levels of FGFR1, FGFR2, FGFR3, FRS2, and AKT in the cortex and hippocampus were measured by western blotting. (b) Densitometric analyses were performed on the immunoreactivities observed in panel a (*n* = 4 mice per group). (c) Levels of p‐FGFR2 and FGFR2 in APPswe‐transfected HT22 cells were detected by western blotting. (d) Densitometric analyses were performed on the immunoreactivities observed in panel c (*n* = 4 replicates per group). (e,f) FGFR2 was activated in the cortex and hippocampus of 3xTg‐AD mice with AAV9‐mediated FGF10 gene delivery (*n* = 6 mice per group). (g–i) The FGFR2 knockdown efficiency was verified by western blotting and RT‐PCR (*n* = 4 replicates per group). (j) Levels of p‐Tau T231, p‐Tau S396, Bcl‐2, Bax, cleaved caspase 3, and β‐Actin in APPswe‐transfected HT22 cells were detected by western blotting. (k) Densitometric analyses were performed on the immunoreactivities observed in panel j (*n* = 4 replicates per group). (l) The potential mechanism diagram involved in FGF10‐mediated protection against AD‐like symptoms and pathologies in both in vivo 3xTg‐AD mice and in vitro APPswe‐transfected HT22 cells. The neuroprotective effects of FGF10 were tested via intranasal delivery and AAV9‐mediated systemic gene delivery methods. FGF10 stimulates the FGFR2/PI3K/AKT signaling pathway, leading to the upregulation of p‐GSK3β S9/ GSK3β and Bcl‐2/Bax. This, in turn, reduces tau hyperphosphorylation and neuronal apoptosis, resulting in protection against neuronal damage and cognitive dysfunction in AD. In in vivo experiments, *p < 0.05 and ***p < 0.001 compared with the 3xTg‐vehicle mice or AAV‐GFP mice. In in vitro experiments, *p < 0.05 and **p < 0.01 compared with APPswe+Sh‐con cells or APPswe+Sh‐con+FGF10 cells. Data are presented as the mean values ± SEM.

In order to elucidate the molecular mechanisms underlying the ameliorative effects of FGF10 on AD‐like symptoms and pathologies, we conducted an analysis of the phosphorylation and total levels of FGFR1, FGFR2, FGFR3, Fibroblast Growth Factor Receptor Substrate 2 (FRS2), and AKT. Our results demonstrated that FGF10 treatment led to increased phosphorylation levels of FGFR2, FRS2, and AKT, while having no significant effect on the phosphorylation levels of FGFR1 and FGFR3 (Figure 7a,b). Moreover, FGFR2 was found to be activated in APPswe‐transfected HT22 cells after FGF10 treatment, as well as in 3xTg‐AD mice following AAV9‐mediated FGF10 gene delivery (Figure 7c–f). These findings suggest that the FGFR2 pathway, rather than FGFR1 or FGFR3 pathways, may play a pivotal role in the neuroprotective effects of FGF10. To further substantiate the involvement of FGFR2 in the neuroprotective effects of FGF10, we performed FGFR2 knockdown experiments in APPswe‐transfected HT22 cells (Figure 7g–i). Notably, FGFR2 knockdown abrogated the opposing effects of FGF10 on tau hyperphosphorylation and apoptosis, as evidenced by our results (Figure 7j,k). Collectively, these data suggest that FGF10 exerts its neuroprotective effects by activating the FGFR2/PI3K/AKT pathway as a key mechanism to ameliorate tau hyperphosphorylation and neuronal apoptosis in 3xTg‐AD mice and APPswe‐transfected HT22 cells.

## 3 DISCUSSION

AD is a chronic neurodegenerative disorder characterized by progressive memory and cognitive decline (Khachaturian, 1985; Mattson, 2004) featuring various important neuropathological alterations such oxidative stress, inflammation, apoptosis, astrocytes reactivity and microglial cells activations, synaptic loss, plasticity changes, and neuronal death (Bamberger & Landreth, 2002; Serrano‐Pozo et al., 2011). Due to the multifactorial nature of AD, FGFs with their wide ranges of neuroprotective activities, have very encouraging therapeutic prospects. FGF10 can inhibit inflammation, apoptosis and oxidative stress and has been found to exhibit neuroprotective effects in numerous CNS disease models (Chen et al., 2017; Fang et al., 2020; Hou et al., 2020; Li et al., 2016). Nevertheless, the protective effects and mechanism of FGF10 on symptoms and pathology in AD have been obscure until now.

In our study, we utilized 3xTg‐AD mice, which are well‐established animal models that exhibit both amyloid and tau pathology, as well as cognitive deficits, to assess the potential therapeutic effect of FGF10 in vivo (Sterniczuk et al., 2010; Stover et al., 2015). Additionally, we employed HT22 cells stably transfected with APPswe, a widely used cell model of AD that displays AD‐like phenotypes, such as SH‐SY5Y, N2a, and HT22 cells (Jiang et al., 2022; Jin et al., 2022; Kong et al., 2020; Li et al., 2020). To determine the correlation between AD and FGF10, we measured FGF10 levels in serum from AD patients, brains of 3xTg‐AD mice, and APPswe‐transfected HT22 cells. Our results revealed reduced FGF10 levels in AD patient serum, 3xTg‐AD mouse brains, and APPswe‐transfected HT22 cells, suggesting that FGF10 may play a crucial role in AD pathogenesis. However, due to the size and biochemical properties of FGF10, its ability to cross the BBB is limited. Therefore, we employed a practical and noninvasive intranasal delivery method that bypasses the BBB to deliver FGF10 to the brain (Chengcheng et al., 2011; Hanson & Frey, 2008). Western blotting and immunofluorescence staining results showed increased FGF10 levels in the cortex and hippocampus of 3xTg‐FGF10 mice, indicating that exogenously administered FGF10 may directly act on these brain regions. However, further studies using other effective methods are warranted to confirm this finding.

Behavioral disturbances, such as depression and anxiety, as well as cognitive dysfunction, are commonly observed in patients with AD (Castellani et al., 2010; Reisberg et al., 1987). Consistent with previous studies, our findings from the open field test, NOR, and MWM also demonstrated cognitive impairments in 3xTg‐AD mice. However, treatment with FGF10 resulted in a significant increase in the percent of time spent in the center of the open field, improved object discrimination index, and reduced escape latency and path length in the Morris water maze, suggesting that FGF10 may alleviate depression, anxiety, and cognitive deficits in AD. The loss of neurons and synapses in the cortex and hippocampus has been shown to strongly correlate with cognitive impairment in AD patients (Serrano‐Pozo et al., 2011). To assess neuronal viability, Nissl staining was performed, and our results showed that FGF10 treatment reduced neuronal damage in 3xTg‐AD mice. Furthermore, we examined the expression of neuronal markers, such as NeuN and MAP2, which are known to play beneficial roles in anti‐apoptotic processes and neuron survival (Zhang et al., 2015), Additionally, synaptic markers PSD95 and synaptophysin, which are associated with synaptic transmission, synaptic plasticity, and synaptogenesis maintenance (Liu et al., 2010), and LTP related molecules phosphorylated and total GluR1 and CaMKII were assessed by western blotting in 3xTg‐AD mice and APPswe‐transfected HT22 cells. Our Nissl staining and western blotting data collectively indicate that FGF10 treatment can ameliorate neuronal damage and synaptic deficits both in vivo and in vitro. While the changes in these biochemical markers may suggest alterations in synaptic function, they do not directly demonstrate functional changes at the synapse. We emphasize that further studies incorporating electrophysiological techniques are needed to validate our findings and provide a more comprehensive understanding of synaptic activity.

The underlying mechanisms of AD are intricate and not yet fully elucidated. However, a growing body of evidence suggests that aberrant accumulation of Aβ, hyperphosphorylation of tau, and apoptosis play pivotal roles in the pathogenesis of AD and have emerged as important targets for AD diagnosis and therapeutic interventions (Behl, 2000; Iqbal et al., 2010; Serrano‐Pozo et al., 2011; Teipel et al., 2022). Dysregulation of Aβ in AD has been shown to activate GSK3β, which subsequently promotes hyperphosphorylation of tau. Hyperphosphorylated tau dissociates from microtubules, leading to impaired axonal transport, formation of NFTs, and subsequent neuronal and synaptic dysfunction (Lauretti et al., 2020; Leroy et al., 2007; Llorens‐Martin et al., 2014). Inhibition of tau kinases, including GSK3β, has been proposed as a potential strategy to reduce NFT aggregation (Toral‐Rios et al., 2020). In our study, we investigated the effects of FGF10 on these key pathological features of AD. Our results revealed that treatment with FGF10 significantly reduced the levels of hyperphosphorylated tau and GSK3β in the cortex and hippocampus of 3xTg‐AD mice. It is worth noting that the activity of GSK3β can be finely regulated by phosphorylation and dephosphorylation at different sites. Autophosphorylation at tyrosine‐279/216 mediates GSK3β activation, while phosphorylation at serine 21/9 by AKT, protein kinases A and B (PKA‐PKB) in the N‐terminus leads to GSK3β inhibition (Lauretti et al., 2020), GSK3β is commonly regulated by inhibitory phosphorylation at Ser9 (Llorens‐Martin et al., 2014). Our findings showed that levels of phosphorylated GSK3β at Ser9 were significantly elevated with FGF10 treatment in 3xTg‐AD mice. In addition to our investigations on the pathological aspects of tau protein, we also conducted extensive research on Aβ pathology. Through in vivo dot blot experiments, we evaluated the expression levels of Aβ1‐40 and Aβ1‐42. Our results revealed a significant upregulation of Aβ1‐40 and Aβ1‐42 in 3xTg‐AD mice compared to wild‐type mice. Notably, intranasal administration of FGF10 and tail vein delivery of AAV‐mFGF10 effectively downregulated the expression of Aβ1‐40 and Aβ1‐42 in the cortex and hippocampus of 3xTg‐AD mice (Figure S5a–f,k–p). We also included the results from ELISA experiments to measure the levels of insoluble and soluble forms of Aβ1–42 and Aβ1–40 in the hippocampus and cortex of 3xTg mice. Our findings indicate that intranasal administration of FGF10 and tail vein injection of FGF10 can improve the expression of soluble and insoluble forms of Aβ1–42 and Aβ1–40 in the hippocampus and cortex of 3xTg mice 3xTg‐AD mice (Figure S5i,j,q,r). In in vitro studies, we employed ELISA to assess the levels of Aβ1‐40 and Aβ1‐42 in the culture supernatant of HT22 cells. In comparison to empty vector‐transfected cells, we observed a significant increase in Aβ1‐40 and Aβ1‐42 expression in APPswe‐transfected HT22 cells. However, treatment with FGF10 successfully reversed this effect, leading to a decrease in Aβ1‐40 and Aβ1‐42 levels (Figure S5g,h). These findings emphasize the potential of FGF10 in counteracting Aβ pathology both in vivo and in vitro.

Neuronal cell loss and synaptic loss in the cortex and hippocampus are major contributors to cognitive decline in AD. Apoptosis has been suggested to be involved in at least some of the neuronal death in AD (Cotman & Anderson, 1995; Shimohama, 2000; Smale et al., 1995). Apoptosis is characterized by mitochondrial dysfunction, leading to activation of caspases, which in turn mediate proteolytic degradation of cytoplasmic and nuclear proteins, nuclear condensation, DNA degradation, and ultimately cell death (Bamberger & Landreth, 2002). Damaged mitochondria release cytochrome c, further activating the caspase family and triggering apoptosis. Caspase‐3, the main executor molecule in multiple apoptotic signaling pathways, is activated during apoptosis. Specifically, it cleaves corresponding cytoplasmic/nuclear substrates, eventually leading to apoptosis. The Bcl‐2/Bax family, a major mitochondrial protein family, acts as a molecular “switch” that initiates apoptosis (Obulesu & Lakshmi, 2014; Sharma et al., 2021). In our study, FGF10 treatment decreased the activity of apoptosis regulators cytochrome c and caspase‐3, and slightly increased the ratio of Bcl‐2 to Bax in the cortices and hippocampi of 3xTg‐AD mice and in APPswe transfected HT22 cells. Similar results were obtained by TUNEL staining in the cortices and hippocampi of 3xTg‐AD mice and in APPswe transfected HT22 cells.

Due to the presence of the BBB, early attempts at CNS disease treatment using AAV were mainly performed through intracranial injections (Huang et al., 2021). However, this method is invasive and provides localized delivery only for diseases known to affect the brain globally (Fischell & Fishman, 2021). Subsequently, systemic injections of AAV9, the first AAV shown to have BBB‐crossing ability in newborn and adult mice, have been widely used in the treatment of various CNS diseases, including PD (Xue et al., 2010), ALS (Foust et al., 2013; Thomsen et al., 2017), HD (Dufour et al., 2014; Vagner et al., 2016) and AD (Yang et al., 2022). We have discovered that intranasal delivery of FGF10 ameliorates AD symptoms and pathologies in mouse and cellular models of AD. However, intranasal delivery requires more frequent administration of FGF10, reducing compliance, which prompted us to search for more effective treatment strategies. AAV9 is an ideal choice for a therapeutic strategy due to its favorable safety profile, strong neural tropism, efficient and persistent gene expression induction with low immunogenicity in brain tissue (Yang et al., 2022). In this study, we tested the therapeutic potential of AAV9‐mediated gene delivery of FGF10 in 3xTg‐AD mice. We found that endogenous FGF10 overexpression attenuated cognitive and synaptic deficits and ameliorated tau hyperphosphorylation and neuronal apoptosis in 3xTg‐AD mice. These results indicate that AAV9‐mediated FGF10 gene transfer could be an effective disease‐modifying therapy for AD.

The PI3K/AKT pathway is a major intracellular signaling pathway responsible for transmitting antiapoptotic signals and controlling neuron survival (Choi & Ho Koh, 2016). GSK3β is one of the key kinases involved in tau phosphorylation, and PI3K/AKT signaling regulates GSK3β by phosphorylating the serine 9 residue, which inhibits its activity (Kitagishi et al., 2014). PI3K/AKT signaling also inactivates Bad, caspase‐3, and caspase‐9, further reducing apoptosis (Franke et al., 2003; Matsuda et al., 2019). However, decreased levels of PI3K subunits and blunted Akt kinase phosphorylation have been observed in AD brains, which are characterized by amyloid‐β and tau pathologies (Gabbouj et al., 2019). FGF10 primarily binds to and activates FGFR2b, leading to activation of the PI3K/AKT pathway (Itoh, 2016; Itoh & Ohta, 2014). Several studies have reported that the FGFR2/PI3K/AKT pathway plays a critical role in CNS diseases, and that FGF10 alleviates spinal cord and cerebral ischemic injuries by activating the PI3K/Akt survival signaling pathway (Chen et al., 2017; Li et al., 2016). In the present study, we observed elevated FGFR2, FRS2, and AKT activation after intranasal FGF10 delivery and AAV9‐mediated FGF10 gene delivery in 3xTg‐AD mice and APPswe‐transfected HT22 cells. We hypothesized that activated FGFR2/PI3K/Akt signaling may play a role in the neuroprotective effects of FGF10. Interestingly, FGFR2 knockdown abolished the opposing effect of FGF10 on tau hyperphosphorylation and its antiapoptotic effect. These results indicate that FGF10 activates the FGFR2/PI3K/Akt pathway as a neuroprotective mechanism to ameliorate tau hyperphosphorylation and neuronal apoptosis in 3xTg‐AD mice and APPswe‐transfected HT22 cells.

We have provided the first evidence of the close relationship between FGF10 and AD. Our study tested the neuroprotective effect of FGF10 in AD using intranasal delivery and AAV9‐mediated systemic gene delivery methods. The results demonstrated that FGF10 improved cognitive functions in 3xTg‐AD mice and ameliorated neuronal damage and synaptic deficits in both 3xTg‐AD mice and APPswe‐transfected HT22 cells. Furthermore, our findings confirmed that FGF10 reduced tau hyperphosphorylation and neuronal apoptosis in 3xTg‐AD mice and APPswe‐transfected HT22 cells. Therefore, therapeutic intervention with FGF10 may hold promise for AD patients. Our results further elucidated potential molecular mechanisms by which FGF10 exerts its protective effects and suggested that regulation of the FGFR2/PI3K/AKT pathway may be a potential target mediating the beneficial effects of FGF10 on AD‐like symptoms and pathologies (Figure 7l). However, further research is necessary to fully understand its mechanism of action and determine its efficacy in human trials.

## 4 MATERIALS AND METHODS

See supplementary information.

## AUTHOR CONTRIBUTIONS

Li Lin, Huiqin Xu, and Kaiming Guo conceived and designed the research. Kaiming Guo, Wenting Huang, Kun Chen, Pengkai Huang, Wenshuo Peng, and Ruiqing Shi conducted the experiments. Tao He, Mulan Zhang, and Hao Wang analyzed the data. Kaiming Guo and Li Lin drafted the manuscript. Jian Hu, Xinshi Wang, and Yangping Shentu provided assistance with the experiments and offered valuable suggestions for improvement.

## CONFLICT OF INTEREST STATEMENT

The authors declare no competing interests.

## Supporting information


Data S1.

## Data Availability

I confirm that my article contains a Data Availability Statement even if no new data was generated (list of sample statements) unless my article type does not require one.
