# Supplementary material for: Fibroblast growth factor 10 ameliorates neurodegeneration in mouse and cellular models of Alzheimer's disease via reducing tau hyperphosphorylation and neuronal apoptosis
Source: Aging Cell. 2023 Jul 28;22(9):e13937. doi: 10.1111/acel.13937 (PMC10497839; doi:10.1111/acel.13937)

**Supplementary Information for**

**Fibroblast growth factor 10 ameliorates neurodegeneration in mouse and cellular models of Alzheimer’s disease via reducing tau hyperphosphorylation and neuronal apoptosis**

Kaiming Guo^1,2^, Wenting Huang^3^, Kun Chen^1,4^, Pengkai Huang^1^, Wenshuo Peng^1,3^, Ruiqing Shi^1^, Tao He^3^, Mulan Zhang^3^, Hao Wang^1,2^, Jian Hu^1^, Xinshi Wang^3^, Yangping Shentu^3^, Huiqin Xu^3*^, Li Lin^1,2,3*^

1. School of Pharmaceutical Sciences, Wenzhou Medical University, University-town, Wenzhou, China.
2. Oujiang Laboratory, Zhejiang Lab for Regenerative Medicine, Vision and Brain Health, Wenzhou, China
3. The First Affiliated Hospital of Wenzhou Medical University, Wenzhou, 325000, China.
4. Jinhua Maternity and Child Health Care Hospital, Jinhua, 321000, China

*** Corresponding Author**

Huiqin Xu, The First Affliated Hospital of Wenzhou Medical University, Wenzhou, China

Email: [xuhuiqin@wmu.edu.cn](mailto:xuhuiqin@wmu.edu.cn)

Li Lin, School of Pharmaceutical Sciences, Wenzhou Medical University, University-town, Wenzhou, China

Email: [linliwz@163.com](mailto:linliwz@163.com)

**This document includes:**

1) Materials and Methods

2) Supplementary Figures and Legends

3) Supplementary Table 1 – Information on human samples

4) Full blots of representative bands

**Materials and Methods**

**Reagents and antibodies**

Recombinant human fibroblast growth factor 10 (rhFGF10) was generously provided by the Key Laboratory of Biotechnology Pharmaceutical Engineering at Wenzhou Medical University, China. The FGF10 ELISA kit (No. SEK10573) was obtained from Sino Biological Inc (Beijing, China). ELISA kits for Aβ1-40 (No. DAB140B) and Aβ1-42 (No. DAB142) were procured from R&D Systems (Minneapolis, MN, USA). The primary antibodies used in this study were as follows: anti-FGF10 (No. ABN44) was procured from Sigma (Sigma-Aldrich, St Louis, MO); anti-NeuN (No. ab177487), anti-PSD95 (No. ab18258), anti-synaptophysin (No. ab32127), anti-MAP2 (No. ab5392), anti-p-Tau T231 (No. ab254409), anti-p-Tau T181 (No. ab151559), anti-p-Tau S396 (No. ab32057), anti-p-Tau S404 (No. ab92676), anti-p-GSK3β S9 (No. ab75814), anti- GluR1 S831 antibody (No. ab109464) and anti-CaMkⅡ antibody (No. ab52476) antibodies were obtained from Abcam (Cambridge, MA, USA); anti-GSK3β (No. 22104-1-AP), anti-cytochrome C (No. 10993-1-AP), anti-Bcl-2 (No. 12789-1-AP), anti-Bax (No. 50599-2-Ig), anti-caspase 3 (No. 19677-1-AP), anti-FRS2 (No. 11503-1-AP), anti-AKT (No. 10176-2-AP), anti-p-AKT S473 (No. 66444-1-Ig), anti-FGFR4 (No. 11098-1-AP), anti-β-actin (No. 66009-1-Ig) and GAPDH (No. 60004-1-Ig) antibodies were purchased from Proteintech (Wuhan, Hubei, China); anti-FGFR1 (No. 9740S), anti-p-FGFR1 Tyr653/654 (No. 52928S), anti-FGFR2 (No. 23328S) antibodies, anti-CaMkⅡ T268 antibody (No. 1271T), anti-Aβ1-40 antibody (No. D8Q71) and anti-Aβ1-42 antibody (No. D9A3A), anti-p-FGFR2 Tyr769 (No. AF8148), anti-FGFR3 (No. AF0160), anti-p-FGFR3 Tyr724 (No. AF8439), and anti-p-FRS2 Tyr436 were obtained from Cell Signaling Technology (Danvers, MA, USA) and Affinity Biosciences (Jiangsu, China), respectively. The anti-GluR1 antibody (No. GTX132945) was purchased from Genetex (Irvine, CA, USA). The secondary antibodies used in this study were goat anti-rabbit immunoglobulin G (IgG) H&L (HRP) (No. ab6721), goat anti-mouse IgG-HRP (No. ab6278), goat anti-rabbit IgG H&L (Alexa Fluor 488) (No. ab150077), and donkey anti-rabbit IgG H&L (Alexa Fluor 647) (No. ab150075), all of which were obtained from Abcam.

**Animals**

In our study, female 3xTg-AD and C57BL/6J mice at the age of 11 months were used. The C57BL/6J mice were obtained from the Animal Center of the Chinese Academy of Science, while the 3xTg-AD mice were obtained from the Shanghai Model Organisms Center. Each mouse was housed individually in a ventilated cage with ad libitum access to food and water, and the temperature was maintained at 25°C. All animal care and experimental procedures were conducted in accordance with the guidelines set forth by the Laboratory Animal Ethics Committee of Wenzhou Medical University.

**FGF10 intranasal administration**

The C57BL/6J and 3xTg-AD mice in our study were administered normal saline or recombinant human FGF10 at a dose of 0.5 mg/kg, dissolved in saline, via intranasal delivery once daily for 21 consecutive days. Following the treatment period, mice in each group underwent a battery of behavioral tests, including open field, novel object recognition, and Morris water maze tests, to assess their behavior. After completion of the behavioral experiments, mice were deeply anesthetized and sacrificed for pathological analyses. All necessary precautions were taken to minimize suffering in accordance with the ethical guidelines established by the Laboratory Animal Ethics Committee of Wenzhou Medical University.

**FGF10 ELISA analysis**

Blood samples were collected from both AD patients and age-matched normal individuals within the age range of 65-85 years. The blood samples were then centrifuged at 2000 ×g for 15 minutes at 4 °C. Subsequently, the serum samples were collected and stored at -80 °C until further examination. The levels of FGF10 were measured using an ELISA analysis, following the procedures outlined in the product introduction provided by the manufacturer.

**Open field test**

Mice were individually placed in an open field arena measuring 60 cm × 60 cm, with environmental noise levels maintained below 50 dB. The central area of the open field was defined as a square measuring 20 cm × 20 cm located in the center of the arena. The mice's movement trajectory, total distance traveled in the open field, and percentage of time spent in the central square were recorded and analyzed using SMART 3.0 software (Panlab Harvard Apparatus, Barcelona, Spain) with video tracking system.

**Novel object recognition test**

Procedures were followed as previously described with some modifications(Li et al., 2022). The novel object recognition test was conducted in a square arena measuring 35 cm × 45 cm × 30 cm. During the habituation period, mice were allowed to freely explore the arena without any objects for 5 minutes. In the training period, two identical square objects (object S1 and S2) were placed in the arena, and mice were allowed to explore for 5 minutes. In the memory testing period, the square object (object S) was replaced with a triangle object (object T). The duration of exploration of familiar objects (ES1 and ES2) during the training period, and the duration of exploration of the familiar object (ES) and novel object (ET) during the memory testing period were recorded. The total exploration time during the training stage was calculated as the sum of ES1 and ES2, and the total exploration time during the memory testing stage was calculated as the sum of ES and ET. The discrimination index (DI) was calculated using the formula: DI = ET / (ES + ET). A discrimination index greater than 0.5 indicated that the mice had a preference for exploring novel objects. The mice's movement trajectory and exploration time towards each object were recorded and analyzed using SMART 3.0 software (Panlab Harvard Apparatus, Barcelona, Spain) with a video tracking system.

**Morris water maze test**

Morris water maze test was followed as previously described(Zhu et al., 2017). The experimental setup consisted of a circular pool with a radius of 50 cm, and a platform with a radius of 6 cm, filled with water maintained at a temperature of 25 ± 1 °C. The tests consisted of two parts: the oriented navigation test and the spatial probe test, which were conducted over a period of five days. The oriented navigation test spanned the first four days, with one session per day consisting of four trials per session. The start positions for the trials were pseudo-randomly varied among the four cardinal points. On the fifth day, the spatial probe test was conducted, during which the platform was removed. The swimming location of the mice was recorded using the DigBehv-MWM Morris computerized tracking system (Shanghai Jiliang Co. Ltd., Shanghai, China). Swimming speed and escape latency were recorded during the oriented navigation test to assess the mice's performance in navigating to the platform. During the spatial probe test, the time spent in the target quadrant and the number of platform crossings were recorded as measures of spatial cognitive functioning.

**Tissue collection**

Brain tissue collection was performed following deep anesthesia with pentobarbital sodium (75 mg/kg dissolved in saline) in animals. Transcardial perfusion with 50 mL of saline was carried out to remove blood from the vasculature. The cortex and hippocampus were carefully dissected from the brains and rapidly frozen in liquid nitrogen for subsequent biochemical assessments. The remaining brain tissues were fixed in 4 % paraformaldehyde for 48 hours, followed by a gradient ethanol dehydration process (70 %, 95 %, and 100 %). Afterward, the fixed brains were treated with xylene and embedded in paraffin. Paraffin-embedded tissues were stored at room temperature until microtome sectioning was performed using a Microm HM325 microtome (Thermo Scientific, USA). For brains that were used for frozen sections with AAV-GFP and AAV-mFGF10, a 48-hour dehydration period in 30 % sucrose was carried out. The brains were then embedded in OCT material and sliced into 20 μm sections using a low temperature thermostat (NX50, Thermo Scientific, USA).

**Western blotting analysis**

Mouse cortex and hippocampal tissues were homogenized using RIPA lysis buffer and the protein concentrations were determined using a bicinchoninic acid (BCA) protein assay kit (20201ES86, Yeasen, Shanghai, China). Equal amounts of protein (60 μg each) were separated by SDS-PAGE and transferred to polyvinylidene fluoride (PVDF) membranes. The membranes were then blocked with 5 % (w/v) skim milk to reduce nonspecific binding and probed with primary antibodies, including NeuN (1:5000), PSD95 (1:1000), MAP2 (1:5000), synaptophysin (1:50000), p-Tau T181 (1:5000), p-Tau T231 (1:5000), p-Tau S404 (1:2000), p-Tau S396 (1:5000), p-GSK3β S9 (1:10000), GSK3β (1:2000), Cytochrome (1:1000), Bcl-2 (1:1000), Bax (1:4000), Caspase-3 (1:1000), FRS2 (1:1000), AKT (1:2000), p-AKT S473 (1:10000), FGFR4 (1:1000), FGFR1 (1:1000), p-FGFR1 Tyr653/654 (1:1000), FGFR2 (1:1000), p-FGFR2 Tyr769 (1:1000), FGFR3 (1:1000), p-FGFR3 Tyr742 (1:1000), p-FRS2 Tyr436, β-actin (1:3000), GluR1 S831 antibody (1:10000), anti-CaMkⅡ antibody (1:10000), anti-GluR1 antibody (1:2000), anti-CaMkⅡ T268 antibody (1:1000), and GAPDH (1:3000).. Following primary antibody incubation, the membranes were incubated with horseradish peroxidase (HRP)-conjugated anti-mouse or anti-rabbit IgG (1:10000). Protein bands were visualized using an enhanced chemiluminescence detection system (P10200, New cell & Molecular Biotech, Suzhou, China), and the immunoreactive bands were quantified using a ChemiDoc XRS+ chemiluminescence detection system (Bio-Rad, CA, USA).

**Immunofluorescent staining**

Deparaffinized sections of 5 μm thickness were rehydrated. Tissues were then blocked with 5% bovine serum albumin (BSA) for 30 minutes to reduce nonspecific binding. After blocking, the sections were incubated overnight with a primary antibody, rabbit anti-FGF10 (1:200). The next day, Alexa-Fluor 488 donkey anti-rabbit secondary antibodies were applied and incubated for 1 hour. The sections were then labeled with DAPI for 7 minutes to stain the nuclei. Finally, the sections were viewed using a Leica confocal microscope (Leica D8IM, Leica, Germany) to capture the images.

**Nissl staining**

Nissl staining was used to detect neuron cell injury. Sections from each group were stained with Nissl staining solution (G1430, Solarbio, Shanghai, China) for 10 minutes at room temperature. After staining, the sections were washed, color-separated, dehydrated, hyalinized, and mounted. In the cortex and hippocampus, neurons with a darkened body and nucleus were classified as damaged neurons, indicating cell injury.

**Plasmids vector construct**

The APP695 (human) fragment was inserted into the pCDH-CMV-MCS-EF1-copGFP-T2A-Puro vector between the BstBⅠ and BamHⅠ restriction sites. The 595th Lys and 596th Met of APP695 were mutated to Asn and Leu, respectively, resulting in the KM595/596NL mutation. The constructed sequence was verified correctly by sequencing. For silencing the expression of FGFR2, a custom-made lentivirus vector carrying shRNA targeting mouse FGFR2 (referred to as Sh-FGFR2) and a control vector Lenti-CMV-EGFP-shRNA (referred to as Sh-con) were purchased from Vector Builder (Guangzhou, China). Additionally, the pAAV-CMV-mFGF10-T2A-EGFP vector (referred to as AAV-mFGF10) and control pAAV-CMV-T2A-EGFP vector (referred to as AAV-GFP) were also obtained from Vector Builder (Guangzhou, China). The pAAV-CMV-mFGF10-T2A-EGFP vector was cloned with the specified mouse FGF10 mRNA sequence (Accession number, NM_009705.3).

**Lentivirus packaging and HT22 cell transduction**

The APPswe and Sh-FGFR2 expression plasmids, along with the control plasmid, were mixed with the packaging plasmids pMD.2G and psPAX, and then transfected into 293T cells. After 72 hours of culture, the cell culture supernatant was collected. The viral titer in the supernatant was determined by RT-PCR. Subsequently, the HT22 cells were transfected with the APPswe lentivirus using different multiplicity of infection (MOI) values of 5, 10, and 20. EGFP green fluorescence was observed 24 hours later to confirm successful transduction. The APPswe-expressing cell line was established after screening with purinomycin, and cells with high levels of APPswe expression were selected using a monoclonal screening method. Similarly, the Sh-FGFR2 lentivirus was transfected into HT22 cells pre-transfected with APPswe, as well as into HT22 cells, using MOI values of 5, 10, and 20. GFP fluorescence was observed 24 hours later to confirm transduction. The efficiency of Sh-FGFR2 knockdown was evaluated by western blotting and RT-PCR.

**Cell treatment**

HT22 cells, along with several stable transfected HT22 cells, were cultured in cell culture plates using Dulbecco Modified Eagle Medium (DMEM) High Glucose, supplemented with 10% fetal bovine serum (FBS) and 1% penicillin-streptomycin. The plates were incubated at 37°C in a humidified environment with 5% CO2. When the cells reached approximately 70% confluence, they were treated with or without recombinant human FGF10 (rhFGF10) at a concentration of 125 ng/mL for a duration of 24 hours. Subsequently, the cells were collected for further examination.

**Cell viability assay**

Cells were seeded in 96-well plates following the instructions provided in the CCK-8 assay kit (C0038, Beyotime, Shanghai, China). Following treatment with various experimental conditions, CCK-8 reagent was added to the cells, and the plates were incubated at 37°C for 4 hours. Subsequently, the absorbance was measured at 450 nm using a Synergy HT microplate reader (Biotek, Vermont, USA) to determine cell viability.

**Immunohistochemistry**

Paraffin pieces (5 μm thick) were rehydrated using graded ethanol to water. Sections were then subjected to antigen retrieval by incubating with 10 mM citrate buffer. Endogenous peroxidase activity was blocked using hydrogen peroxide solution. Rabbit anti-Tau T231 primary antibodies (dilution 1:200) were applied to the sections and incubated. Slides were developed using a liquid substrate containing diaminobenzidine, followed by counterstaining with hematoxylin and mounting with neutral resins. Images were captured using a Nikon light microscope (Nikon 80i, Nikon, Tokyo, Japan).

**TUNEL assay**

Neuronal apoptosis in the cortex and hippocampus of various experimental groups was identified using the TUNEL (Terminal deoxynucleotidyl transferase dUTP Nick End Labeling) assay kit (C1089, Beyotime, Shanghai, China) according to the manufacturer's instructions. Briefly, paraffin-embedded brain sections (5 μm) were deparaffinized and rehydrated. Proteinase K solution was applied to penetrate the slices for 30 minutes at room temperature, followed by incubation in a humidified chamber for 30 minutes at 37 °C with reaction buffer containing terminal deoxynucleotidyl transferase. Nuclei were faintly stained with DAPI. Confocal microscopy (Leica DMI8, Leica, Germany) was used to examine the sections and identify TUNEL-positive cells. In cellular experiments, cells were stained with the TUNEL assay kit according to the manufacturer's protocol after relevant treatment. The percentage of TUNEL-positive cells was quantified using confocal microscopy (Leica DMI8, Leica, Germany).

**Flow cytometric analysis of ROS**

DHE fluorescent probe (CA1420, Solarbio, Beijing, China) was utilized to detect ROS in HT22 cells and APPswe-transfected HT22 cells. The cells were harvested and then incubated with 10 mM DHE for 30 minutes at 37°C, following the manufacturer's instructions. DHE is a commonly used probe that reacts specifically with superoxide anion (O^2-^) to form ethidium, which exhibits red fluorescence upon intercalation with DNA. After the incubation period, the red fluorescence emitted by ethidium was measured using a Beckman Coulter Cytoflex flow cytometer (Beckman Coulter, Brea, CA, USA). The flow cytometer was set to excite DHE at a wavelength of 535 nm, which is the optimal excitation wavelength for DHE, and measure the emitted fluorescence at a wavelength of 610 nm. This allows for quantitative measurement of ROS levels in the cells, as the intensity of the red fluorescence is proportional to the amount of ROS present in the cells.

**AAV production and purification**

The production and purification of recombinant AAV particles followed Addgene protocols, which can be found online at <https://www.addgene.org/protocols/aav-production-hek293-cells/> and <https://www.addgene.org/protocols/aav-purification-iodixanol-gradient/>. The AAV particles were produced in 293T cells that were cultured in DMEM with High Glucose, 10 % fetal bovine serum, and 1 % penicillin/streptomycin. The cells were split every 3-4 days using 0.25 % trypsin. To produce replication-incompetent AAV particles, three different plasmids were co-transfected into 293T cells using polyethylenimine (PEI). These plasmids included a transgene-containing plasmid, a packaging plasmid (pAAV-RC9) containing the rep and cap genes, and a helper plasmid (pHelper) containing three adenoviral helper genes. After transfection, cells and supernatant were harvested at 120 hours. The cells were harvested and viral particles present in the supernatant were concentrated by precipitation with 8% Polyethylene glycol 8000 (PEG8000). The lysate was then clarified by centrifugation at 5000g for 15 minutes to remove cellular debris. The viral phase was isolated by iodixanol step gradient (15 %, 25 %, 40 %, and 60 %) with the AAV particles concentrated in the 40 % fraction. The concentrated viral particles were then further purified by PBS with 100K cut-off concentrator. Virus titers were determined by qPCR using the procedure provided by Addgene.

**AAV tail-vein injection**

For tail-vein injections of AAV vectors, mice were placed into a restrainer to immobilize them. The tail was warmed in water for 30 seconds to increase blood flow, and then wiped with 70% isopropyl alcohol pads to sterilize the injection site. A volume of 150 μL of vector solution (6.2×10^11 viral genomes/mouse) was slowly injected into a lateral tail vein using a syringe and needle, taking care to avoid injecting air bubbles. After the injection, the injection site was gently finger-clamped to prevent bleeding, and pressure was applied until bleeding stopped.

**Frozen sections** **immunofluorescent staining**

Frozen sections were subjected to immunofluorescent staining. Brain tissue embedded in OCT compound was washed three times with PBS, followed by blocking with 5 % BSA for 1 hour at room temperature. Subsequently, tissue sections were incubated with primary antibody p-Tau T231 (dilution 1:250) in PBS containing 1 % BSA at 4°C overnight. The next day, sections were incubated with the secondary antibody for 1 hour at room temperature in the dark. Nuclei were counterstained with DAPI, and images were acquired using a confocal microscope (Leica D8IM, Leica, Germany).

**RNA extraction and RT-PCR**

Total RNA was extracted from APPswe-transfected HT22 cells by TriPure Isolation Reagent (Roche, South San Francisco, CA, USA) according to the operating instructions. RNA concentrations were quantified by Nanodrop spectrometry (Thermo Fisher Scientific, MA, USA) and guaranteed the OD260/OD280 value is within 1.8-2.1. PrimeScript RT Reagent Kit (RR037A, TaKaRa, Japan) was used to synthesize cDNA and reverse transcription was conducted with SYBR Green PCR Master Mix (BioRad, Hercules, CA, USA) in 10 μL final reaction volume. Forward and reverse primer sequences of FGFR2 used in RT-PCR were GCTATAAGGTACGAAACCAGCAC and GGTTGATGGACCCGTATTCATTC respectively, were purchased from Sangon Biotech (Shanghai, China). Using PCR program that included 40 cycles of denaturation, annealing, and extension. The expression levels of the target gene were normalized to the expression level of β-actin, and the results were calculated using the 2^-ΔΔCT^ method. The results are presented as the mean ± SEM of duplicate samples from three independent experiments.

**Rotarod test**

The rotarod test assesses the motor performance of mice by measuring their ability to remain on an accelerating rotating rod. Female 11-months aged 3xTg-AD and C57BL/6J mice initially underwent a three-day adaptation trial at a constant speed of 20 rpm/min. Subsequently, they were subjected to four acceleration trials where the speed increased from 4 to 40 rpm within 5 minutes. There was a 30-minute interval between each trial, and the average retention time was recorded.

**Visual cliff avoidance test**

The visual cliff avoidance experiment was conducted following a modified protocol to assess visual function(Glynn, Bortnick, & Morton, 2003; Tzameret et al., 2019). To conduct the visual cliff avoidance experiment, we used an open-top and transparent box. The dimensions of the box were 60×60 cm with a height of 30 cm. During the experiment, the box was placed at the edge of the laboratory table, with half of the box's bottom covering the table ("table side"), while the other half was suspended at the edge of the table, approximately 80 cm above the ground ("cliff side"), creating a virtual cliff. To highlight the cliff's edge, a checkerboard pattern was placed on the table side of the box and on the floor beneath it. Throughout the experiment, the female 11-months aged 3xTg-AD and C57BL/6J mice were consistently placed on a platform (10×7×2 cm) positioned at the center of the transparent box, facing the side with the table. The indoor light intensity at the level of the mice's eyes was measured using a luminance meter and recorded as 29.7 cd/m^2^. The mice's activity on the platform was recorded for a duration of 2 minutes. Several parameters were analyzed, including the latency to leave the starting platform and the direction (cliff or table) of the first foot when leaving the platform.

**Visible platform Morris water maze test**

The visible platform Morris water maze test was conducted using an experimental setup comprising a circular water pool with a radius of 50 cm and a platform with a radius of 6 cm. The pool was filled with water and maintained at a constant temperature of 25±1℃. The visible platform training spanned across 3 days. To enable the mice to see the platform, a black label was attached to the platform, facilitating its visibility. Each day, the mice underwent a total of 4 trials, with the platform being placed in different locations for each trial. This arrangement aimed to teach the mice to identify the location of the visible platform. If a mouse failed to locate the platform within the designated time, it was immediately guided to the platform manually. Once a mouse successfully reached the platform, it remained on the platform for a duration of 15 seconds before being removed from the water maze. During the test, the DigBehv-MWM Morris computerized tracking system (Shanghai Jiliang Co. Ltd., Shanghai, China) was utilized to record and analyze parameters such as swimming speed and escape latency, providing quantitative data for further analysis and interpretation.

**Dot blot immunoassay**

Dot lot Immunoassay performed as previously described with some modifications(Ng et al., 2021). The protein concentration of cortical lysates and hippocampal lysates was determined using the BCA assay kit. The lysates were diluted to a concentration of 1 μg/μL. A total of 2 microliters of each sample were spotted onto a nitrocellulose membrane. The membrane was then air-dried at room temperature for 1 hour. Next, the membrane was blocked with 5% non-fat milk/TBST solution for 1 hour to prevent non-specific binding. After the blocking step, the membrane was incubated with rabbit anti-Aβ1-42(dilution 1:1000) and rabbit anti-Aβ1-40 (dilution 1:1000) antibodies, targeting specific Aβ peptides, for a designated period of time. Following the primary antibody incubation, the membrane was incubated with an HRP-conjugated rabbit anti-mouse IgG secondary antibody for 1 hour. This secondary antibody binds to the primary antibodies, allowing for the detection of the target Aβ peptides. To visualize the signal, the Western bright Quantum HRP substrate was applied to the membrane. The substrate reacts with the HRP enzyme, resulting in a chemiluminescent signal. The signal was captured using the ChemiDoc Imaging system (Bio-Rad, USA) for further analysis and quantification.

**Aβ1-42 and Aβ1-40 ELISA analysis**

Brain tissue was homogenized in 300 µL of RIPA buffer and sonicated to extract both soluble and insoluble Aβ. After centrifugation, the resulting supernatants were carefully collected and stored at -80°C for further analysis. To measure the levels of insoluble Aβ, the pellet obtained from centrifugation was dissolved in guanidine-HCl and appropriately diluted with assay buffer. The concentrations of soluble and insoluble forms of Aβ1–42 and Aβ1–40 in the brain tissue samples were determined and expressed as ng/mg of total protein. Empty vector-transfected and APPswe-transfected HT22 cells were seeded into 6-well plates. Following cell inoculation, the cells were treated with rhFGF10 (125 ng/mL) for a duration of 24 hours. After the treatment period, both the culture supernatants and cells were collected and subjected to centrifugation at 1000 rpm for 10 minutes using a bench-top centrifuge and resulting supernatants were utilized. The levels of Aβ1–42 and Aβ1–40 in all samples were measured following the protocols provided by the ELISA manufacturer.

**Statistical analysis**

Data was analyzed by GraphPad Prism software (GraphPad Software, Inc., San Diego, CA). The results are presented as the mean ± standard error of the mean (SEM). Statistical differences among data from three and more groups were performed using analysis of variance (ANOVA) followed by Tukey's test. Comparisons between two groups were performed using independent Student’s t tests. For all tests, P < 0.05 was considered statistically significant.

**Fig. S1**


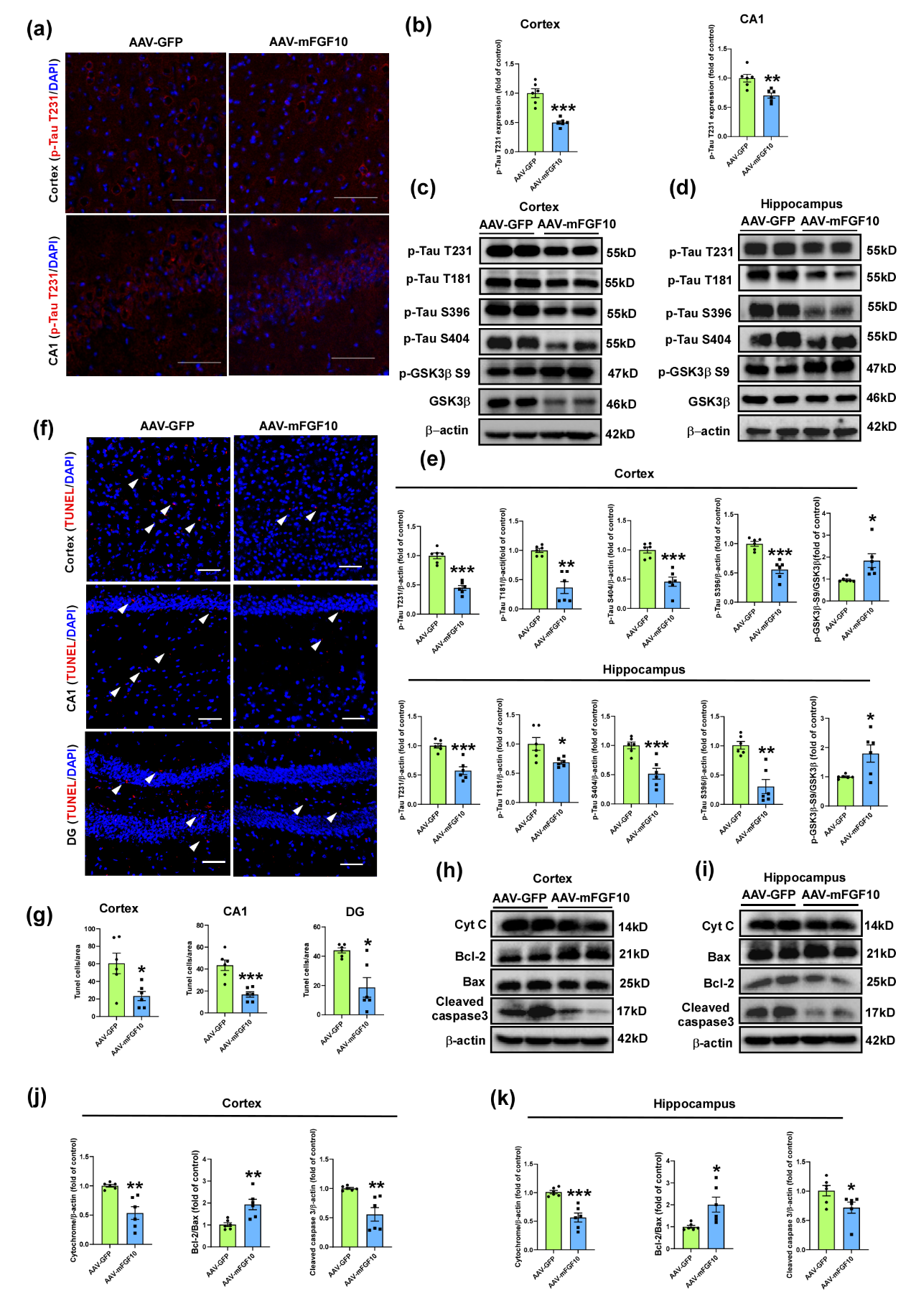


**Fig.** **S1. Endogenous FGF10 overexpression ameliorates tau hyper-phosphorylation and neuronal apoptosis in 3xTg-AD mice.** (a) Immunofluorescence staining was performed to evaluate the expression of p-Tau T231 in the cortex and hippocampus (CA1) of mice. Scale bar, 100 µm. (b) Quantification of immunofluorescence staining in panel a (n = 6 mice per group). (c-d) Western blotting was performed to detect the levels of p-Tau T231, p-Tau T181, p-Tau S396, p-Tau S404, GSK3β, and p-GSK3β S9 in the cortex and hippocampus of 3xTg-AD mice. (e) Densitometric analyses were performed on the immunoreactivities observed in panels c and d (n = 6 mice per group). (f) TUNEL assay was used to evaluate neuronal apoptosis in the cortex and hippocampus (CA1 and DG) of 3xTg-AD mice, with white arrows indicating TUNEL-positive cells. Scale bar, 100 µm. (g) Quantification of TUNEL staining in panel a (n = 6 mice per group). (h-i) Western blotting was performed to detect the levels of cytochrome c (abbreviated to Cyt C), Bcl-2, Bax, and cleaved caspase 3 (active form of caspase 3), and β-actin in the cortex and hippocampus. (j-k) Densitometric analyses were performed on the immunoreactivities observed in panels h and i (n = 6 mice per group). P < 0.05, ** P < 0.01, and *** P < 0.01 compared with the AAV-GFP mice. Data are presented as the mean values ± SEM.

**Fig. S2**


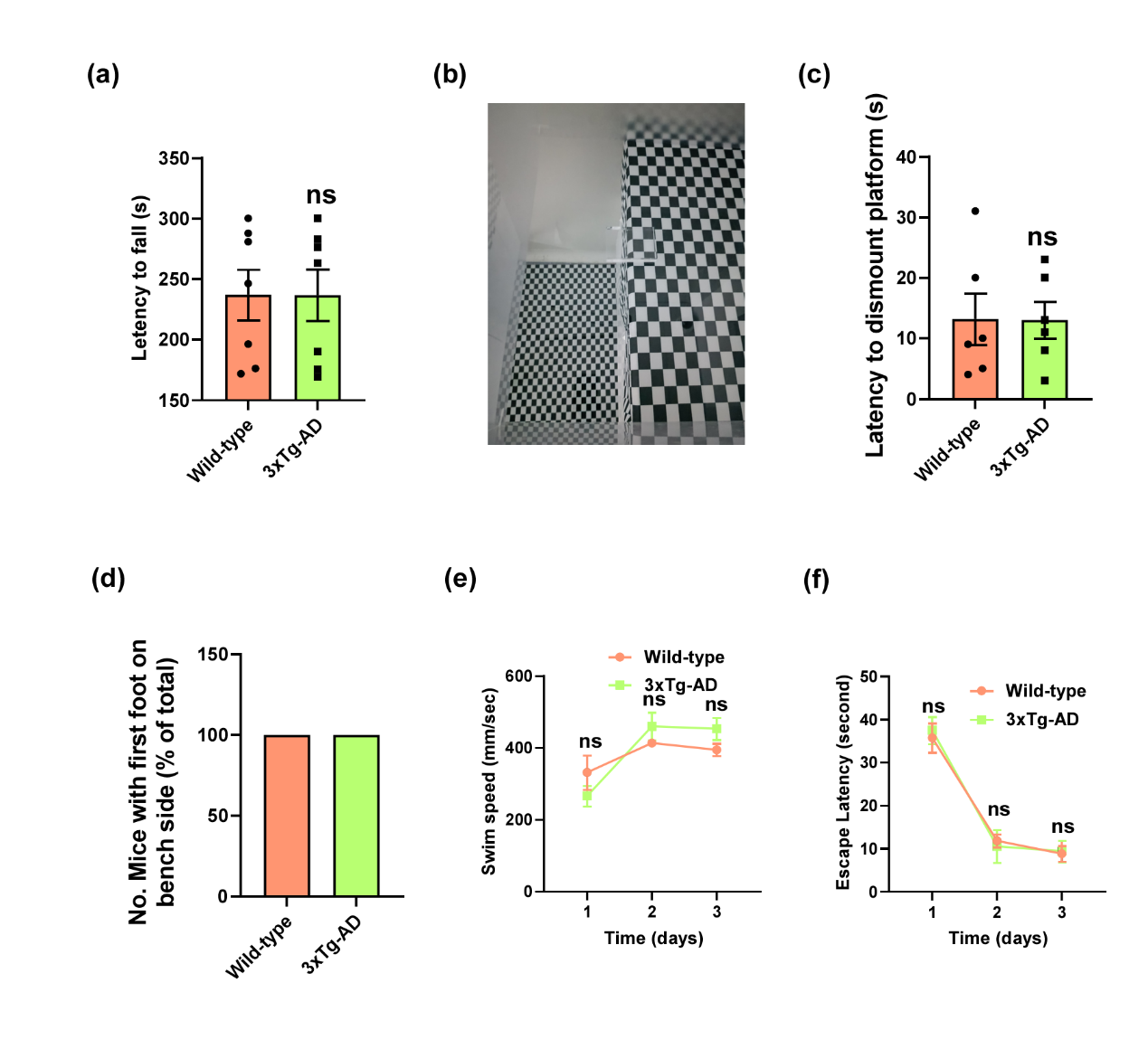


**Fig. S2. Motor, visual, and motivation functions were not impaired in 11-month-old 3xTg-AD mice.** (a) Motor function was evaluated using the Rotarod test (n = 7 mice per group), (b) visual function was assessed using the Visual Cliff avoidance test apparatus. (c) The latency to dismount the starting stage was measured in both 11 months aged wild-type and 3xTg-AD mice (n = 7 mice per group). (d) The percentage of mice that successfully took the first footstep over the bench side was determined (n = 7 mice per group). Motivation function was evaluated through the Visible Platform water maze test and average swim speed (e) and escape latency (f) were recorded and analyzed. The results were compared between 3xTg-AD and wild-type mice. Data are presented as mean values ± SEM.

**Fig. S3**


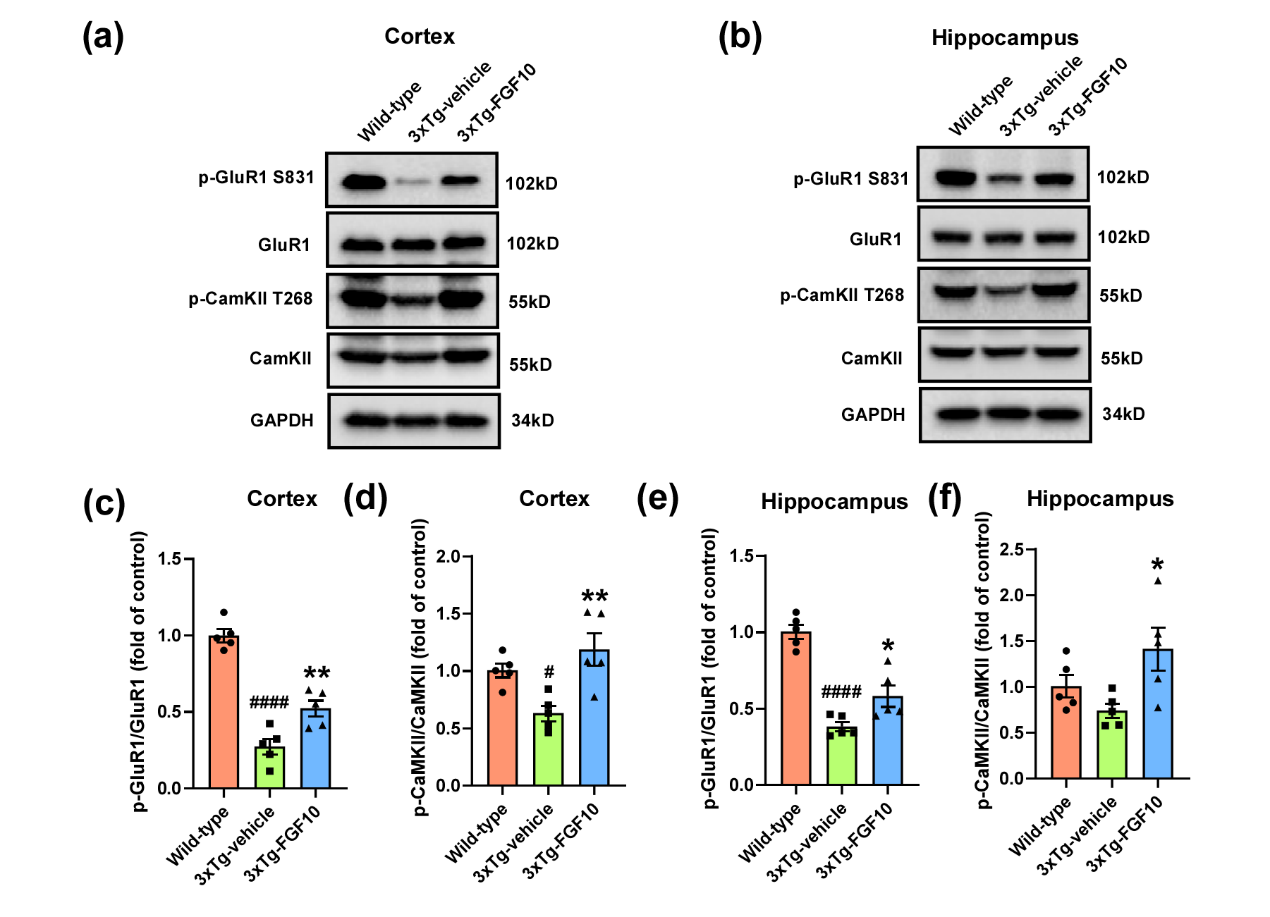


**Fig. S3. Rescue of impaired LTP in 3xTg-AD mice following intranasal administration of FGF10.** (a-b) Western blot analysis was performed to evaluate the levels of GluR1,p-GluR1 S831, CaMKII and p-CaMKII T286 in the cortex and hippocampus of both wild-type and 3xTg-AD mice. (c-f) Densitometric analyses were conducted on the immunoreactivities observed in panels a and b (n = 5 mice per group). # P < 0.05 and #### P < 0.0001 compared with the wild-type mice; * P < 0.05 and ** P < 0.01 compared with the 3xTg-vehicle mice. Data are presented as mean values ± SEM.

**Fig. S4**

**
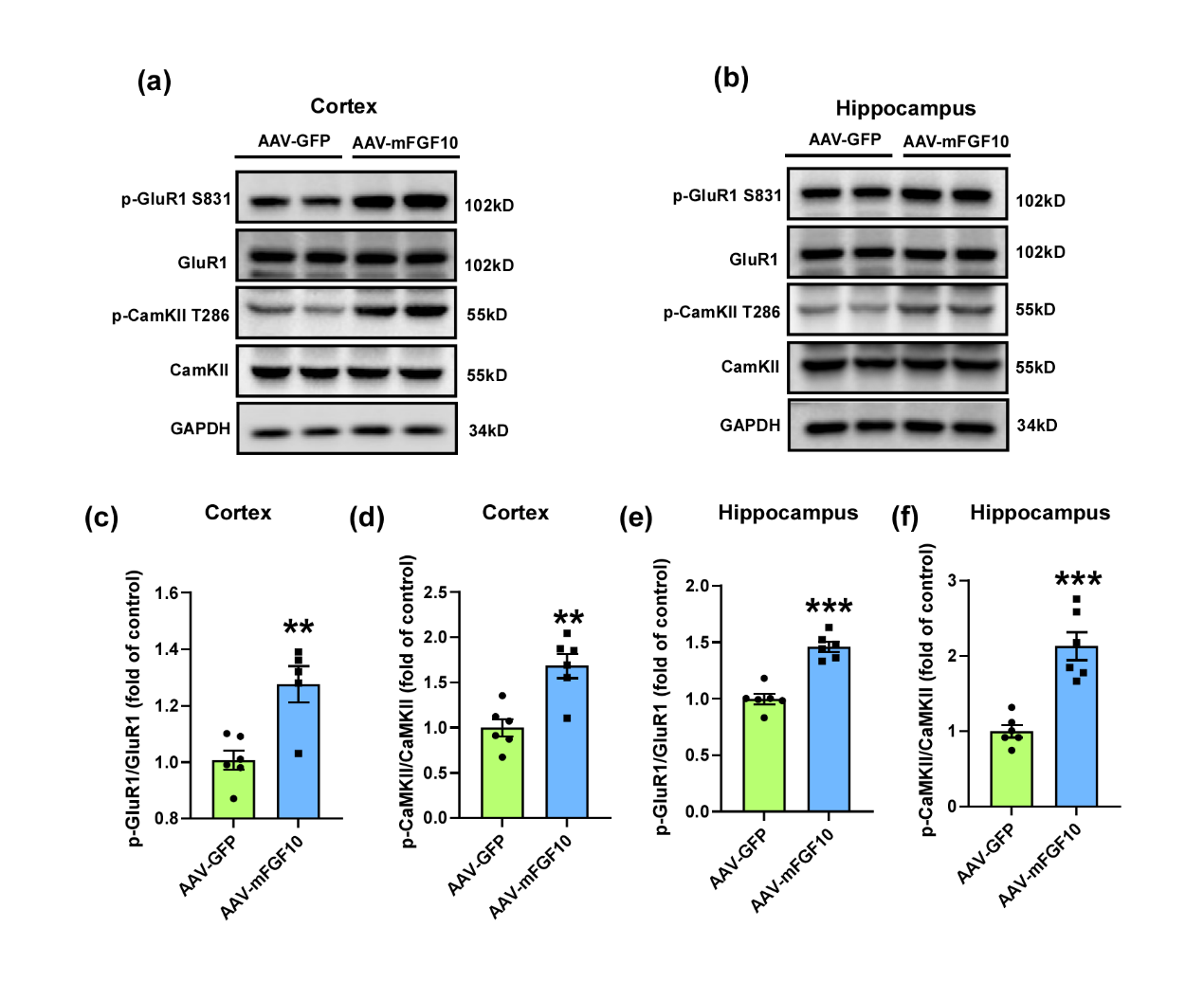
**

**Fig. S4. Endogenous FGF10 overexpression restores impaired LTP in 3xTg-AD mice.** (a-b) Levels of the CaMKII, p-CaMKII T286, GluR1, and p-GluR1 S831 in the cortex and hippocampus of 3xTg-AD mice were detected by Western blotting. (c-f) Densitometric analyses were performed on the immunoreactivities observed in panels a and b (n = 5-6 mice per group). ** P < 0.01 and *** P < 0.001 compared with the AAV-GFP mice. Data are presented as mean values ± SEM.

**Fig. S5**

**
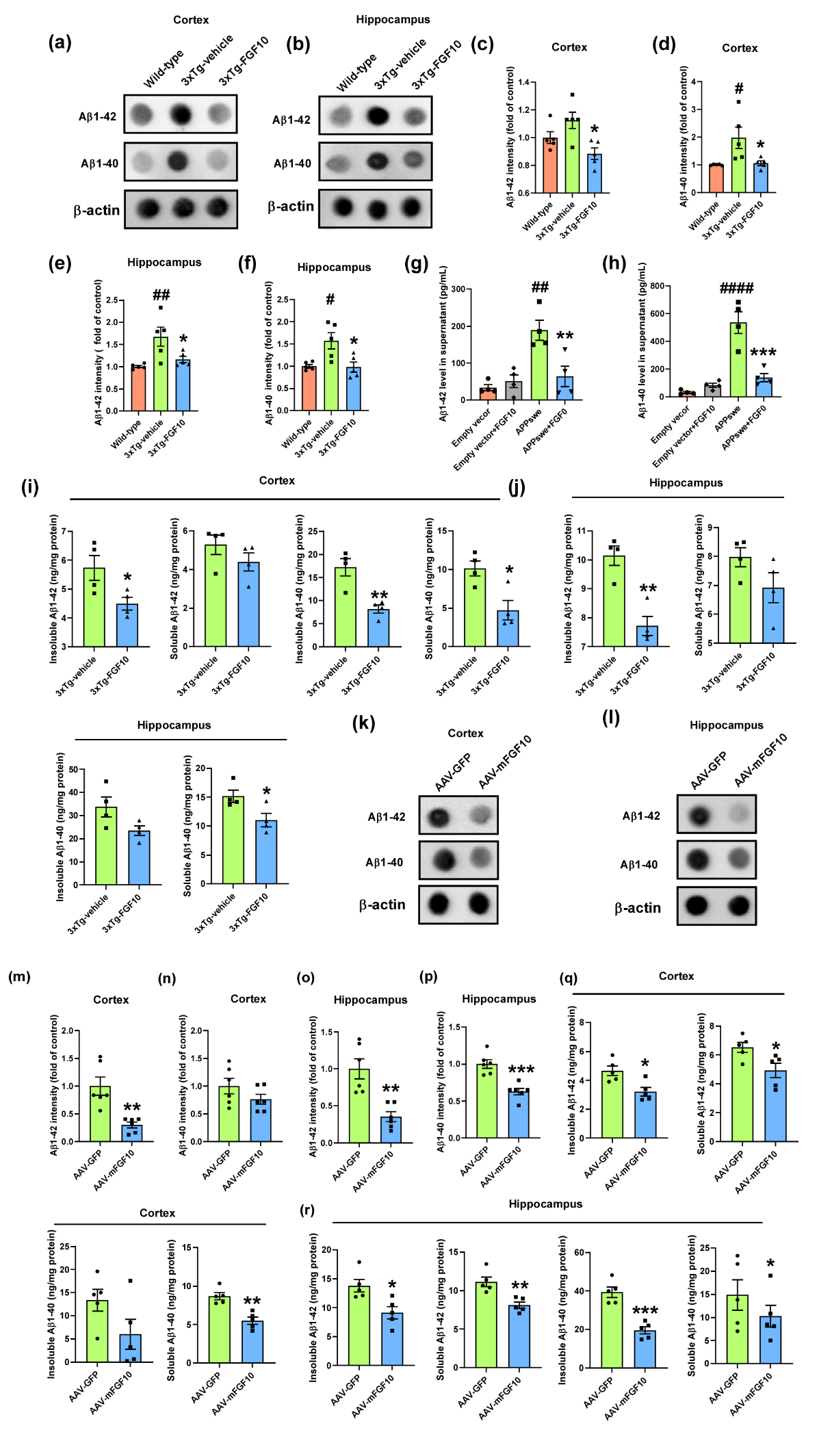
**

**Fig. S5. FGF10 treatment reduces Aβ1-42 and Aβ1-40 levels in vivo and in vitro.**

(a-b) Dot-blot analysis of Aβ1-42 and Aβ1-40 in the cortex and hippocampus of wild-type and 3xTg-AD mice with intranasal administration of FGF10. (c-f) Densitometric analyses were performed on the immunoreactivities observed in panels a and b (n = 5 mice per group). # P < 0.05 and ## P < 0.01 compared to wild-type mice; * P < 0.05 compared to 3xTg-vehicle mice. Data are presented as mean ± SEM. Aβ1-42 (g) and Aβ1-40 (h) levels in HT22 cell supernatant were detected using ELISA (n = 4 replicates per group). ## P < 0.01 and #### P < 0.0001 compared to empty vector cells; ** P < 0.01 and *** P < 0.001 compared to APPswe cells. Data are presented as mean ± SEM. (i-j) Insoluble and soluble forms of Aβ1–42 and Aβ1–40 in the cortex and hippocampus of 3xTg-AD mice with intranasal administration of FGF10 were assayed by ELISA (n = 4 mice per group). * P < 0.05 and ** P < 0.01 compared to 3xTg-vehicle mice. Data are presented as mean ± SEM. (k-l) Dot-blot analysis of Aβ1-42 and Aβ1-40 in the cortex and hippocampus of 3xTg-AD mice with endogenous FGF10 overexpression. (m-p) Quantification analysis of Aβ1-42 and Aβ1-40 levels in the cortical and hippocampal lysates of 3xTg-AD mice (n = 6 mice per group). (q-r) Insoluble and soluble forms of Aβ1–42 and Aβ1–40 in the cortex and hippocampus of 3xTg-AD mice with endogenous FGF10 overexpression were assayed by ELISA (n = 5 mice per group). * P < 0.05, ** P < 0.01 and *** P < 0.001 compared to AAV-GFP mice. Data are presented as mean ± SEM.

**Reference**

Glynn, D., Bortnick, R. A., & Morton, A. J. (2003). Complexin II is essential for normal neurological function in mice. *Hum Mol Genet, 12*(19), 2431-2448. doi:10.1093/hmg/ddg249

Li, S., Zhou, Q., Liu, E., Du, H., Yu, N., Yu, H., . . . Wang, J. Z. (2022). Alzheimer-like tau accumulation in dentate gyrus mossy cells induces spatial cognitive deficits by disrupting multiple memory-related signaling and inhibiting local neural circuit. *Aging Cell, 21*(5), e13600. doi:10.1111/acel.13600

Ng, R. C., Jian, M., Ma, O. K., Bunting, M., Kwan, J. S., Zhou, G. J., . . . Chan, K. H. (2021). Chronic oral administration of adipoRon reverses cognitive impairments and ameliorates neuropathology in an Alzheimer's disease mouse model. *Mol Psychiatry, 26*(10), 5669-5689. doi:10.1038/s41380-020-0701-0

Tzameret, A., Sher, I., Edelstain, V., Belkin, M., Kalter-Leibovici, O., Solomon, A. S., & Rotenstreich, Y. (2019). Evaluation of visual function in Royal College of Surgeon rats using a depth perception visual cliff test. *Vis Neurosci, 36*, E002. doi:10.1017/S095252381800007X

Zhu, X., Wang, S., Yu, L., Jin, J., Ye, X., Liu, Y., & Xu, Y. (2017). HDAC3 negatively regulates spatial memory in a mouse model of Alzheimer's disease. *Aging Cell, 16*(5), 1073-1082. doi:10.1111/acel.12642

**Supplementary Table 1 – Information on human samples**

| **Patient ID** | **Age** | **Gender** | **Clinical Diagnosis** | **Disease Severity** |
| --- | --- | --- | --- | --- |
| AD001 | 75 | M | AD | Moderate |
| AD002 | 82 | M | AD | Moderate |
| AD003 | 67 | F | AD | Mild |
| AD004 | 80 | F | AD | Severe |
| AD005 | 72 | F | AD | Mild |
| AD006 | 76 | M | AD | Moderate |
| AD007 | 82 | M | AD | Moderate |
| AD008 | 68 | M | AD | Moderate |
| AD009 | 82 | F | AD | Moderate |
| AD010 | 85 | M | AD | Severe |
| AD011 | 75 | M | AD | Moderate |
| AD012 | 78 | F | AD | Moderate |
| AD013 | 78 | F | AD | Moderate |
| AD014 | 85 | M | AD | Severe |
| AD015 | 80 | F | AD | Severe |
|  |  |  |  |  |
| **Nomal ID** | **Age** | **Gender** | **Clinical Diagnosis** | **Disease Severity** |
| NO001 | 72 | F | / | / |
| NO002 | 78 | M | / | / |
| NO003 | 81 | M | / | / |
| NO004 | 76 | M | / | / |
| NO005 | 83 | F | / | / |
| NO006 | 80 | M | / | / |
| NO007 | 81 | F | / | / |
| NO008 | 65 | M | / | / |
| NO009 | 68 | F | / | / |
| NO010 | 79 | M | / | / |
| NO011 | 82 | M | / | / |
| NO012 | 82 | F | / | / |
| NO013 | 77 | M | / | / |
| NO014 | 78 | F | / | / |
| NO015 | 76 | F | / | / |

**Full blots of representative bands:**


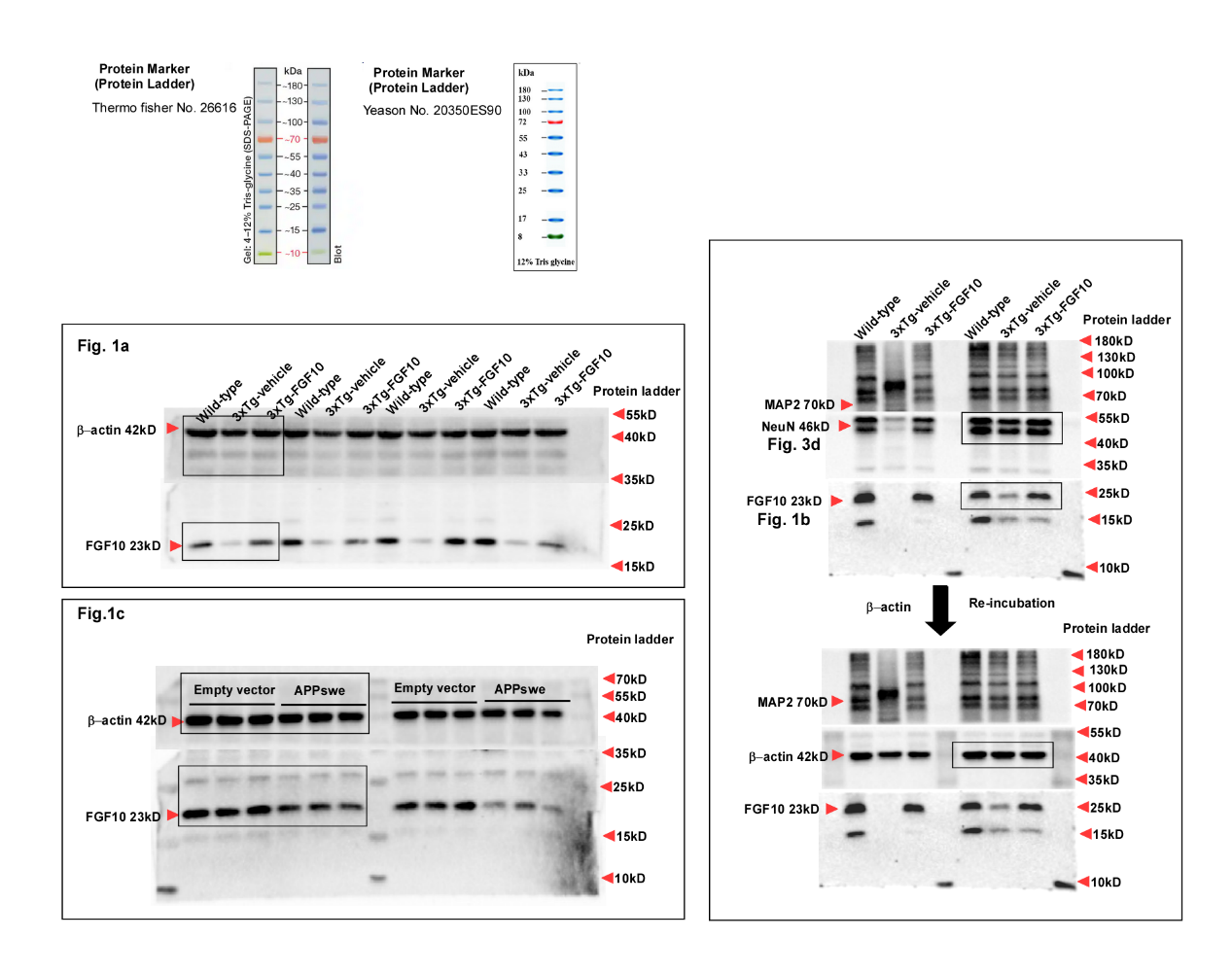


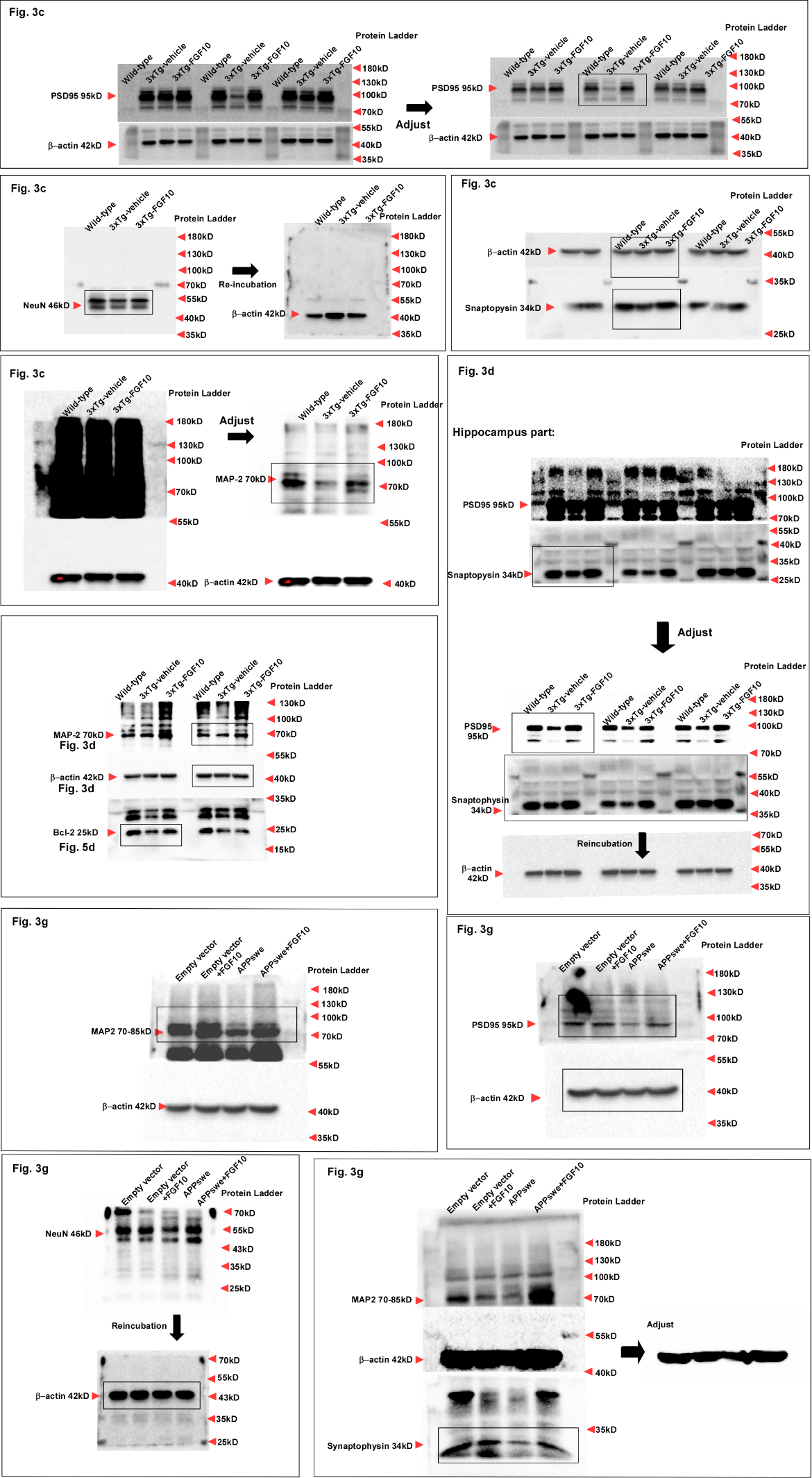


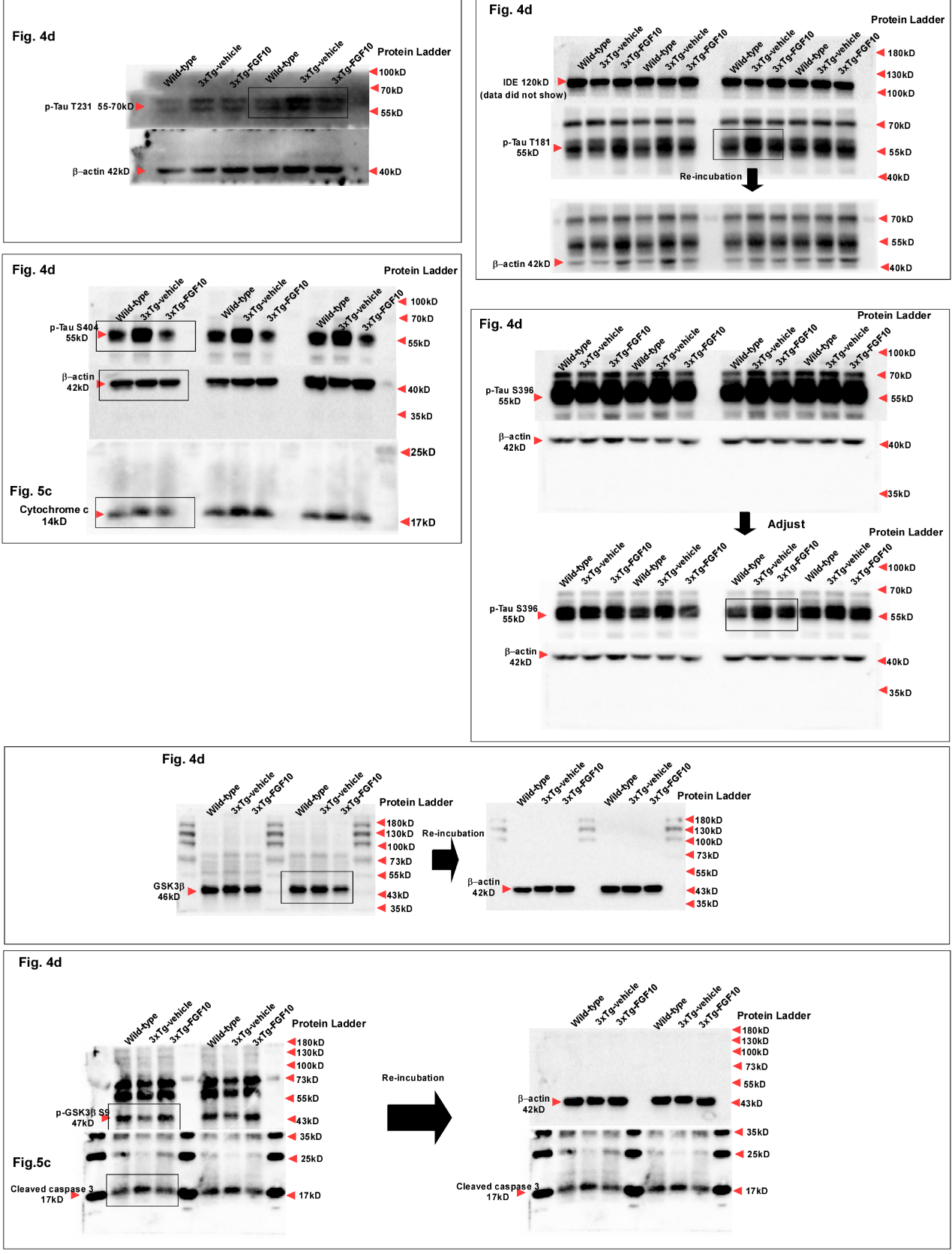


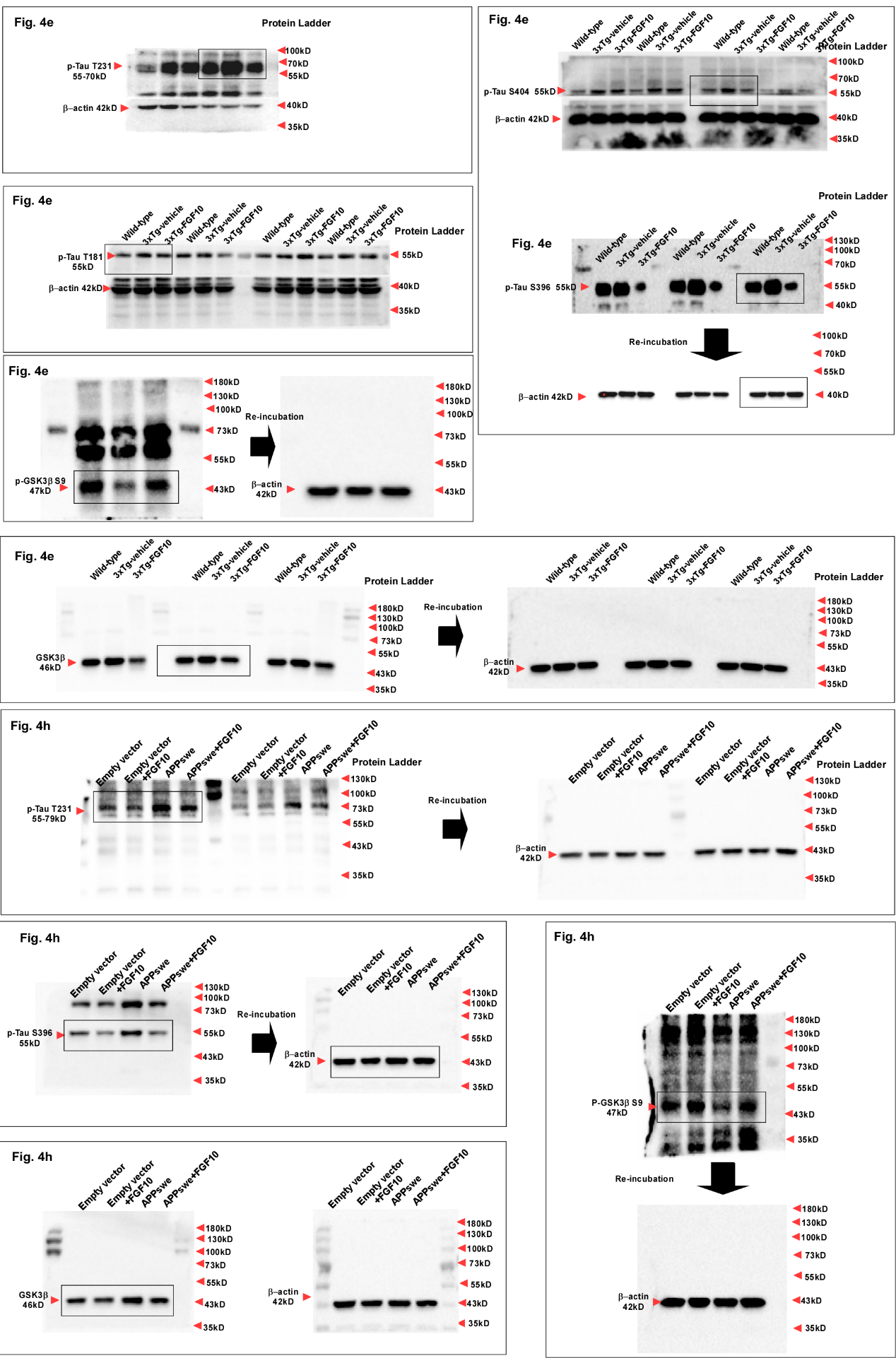


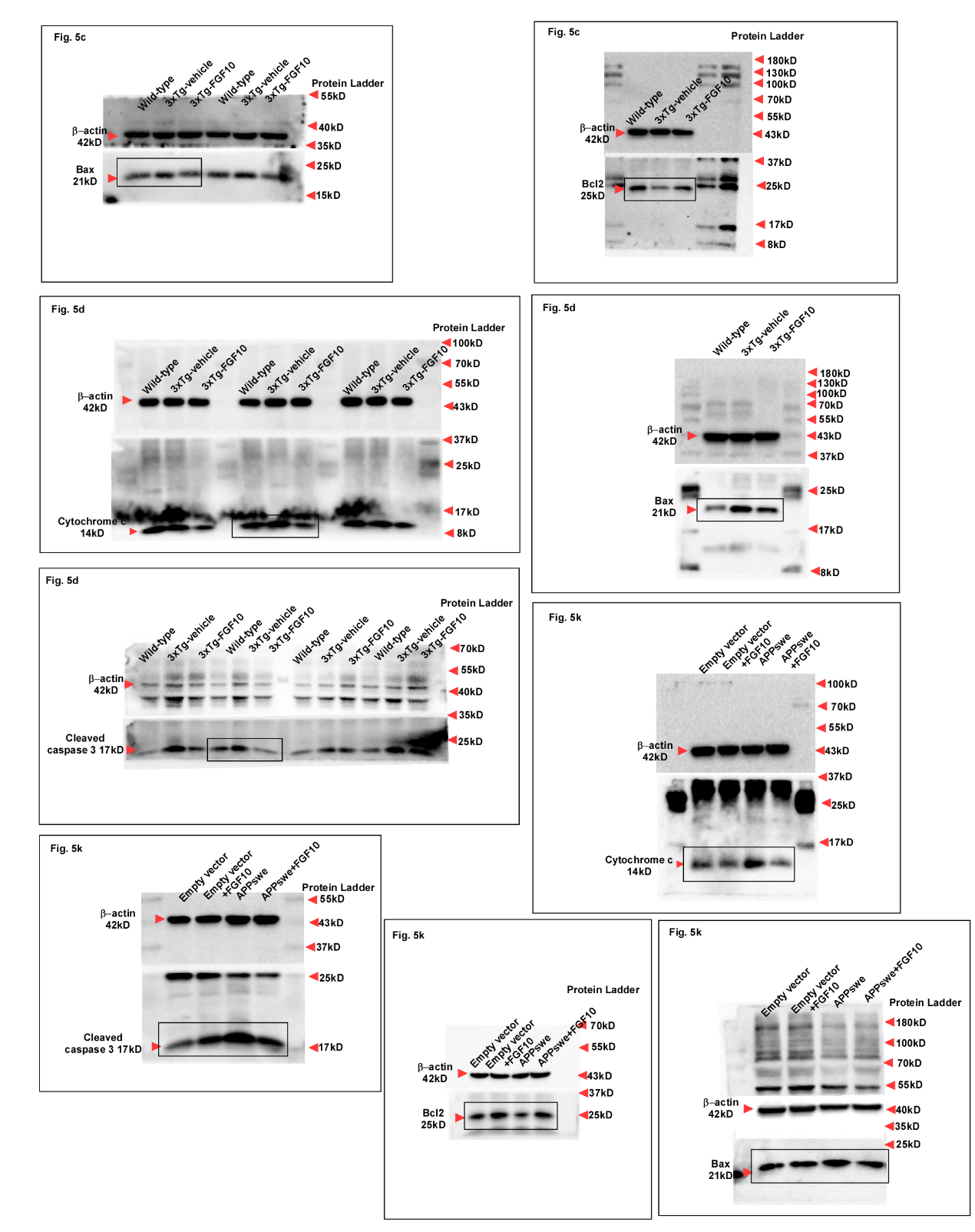


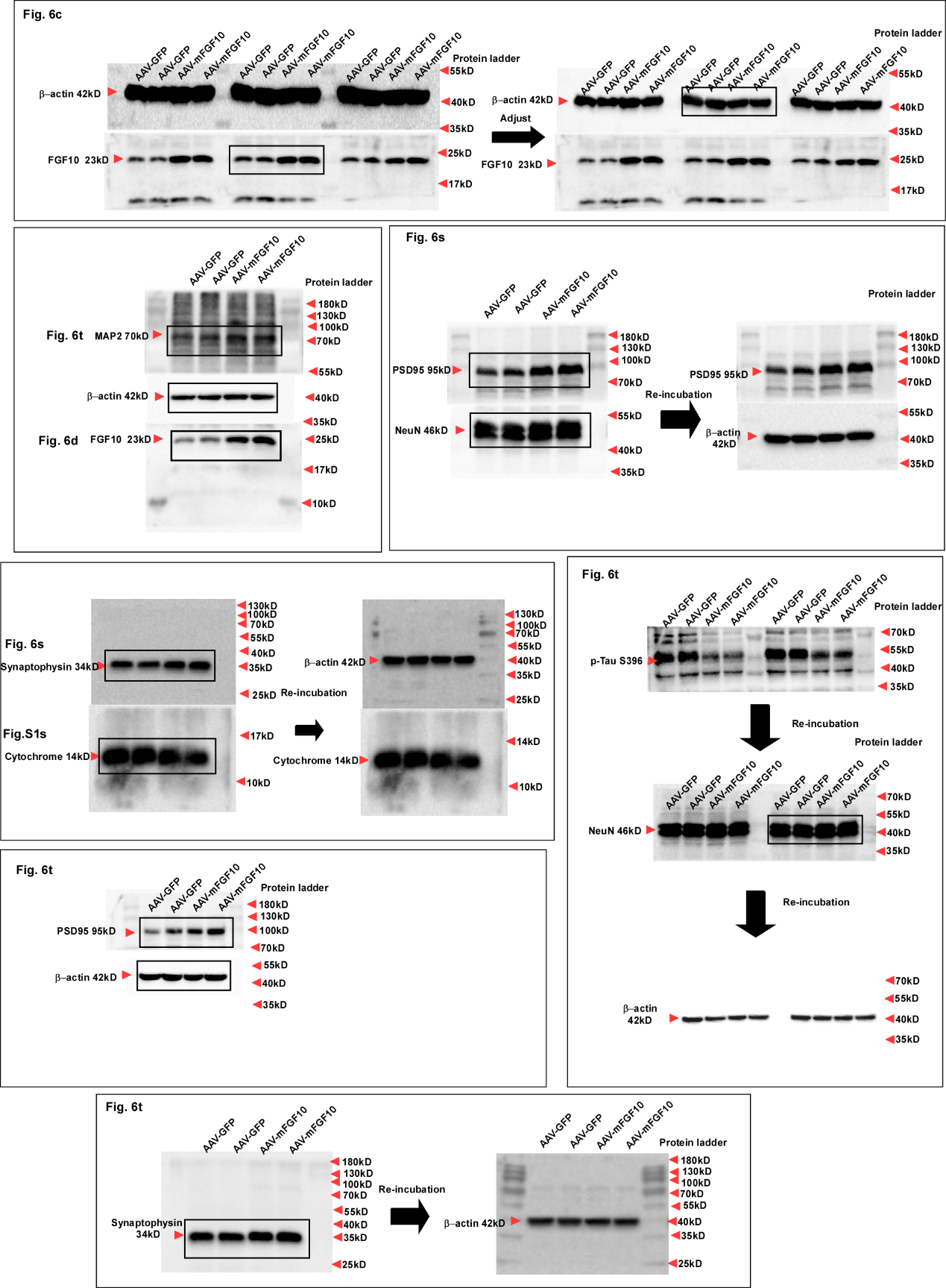


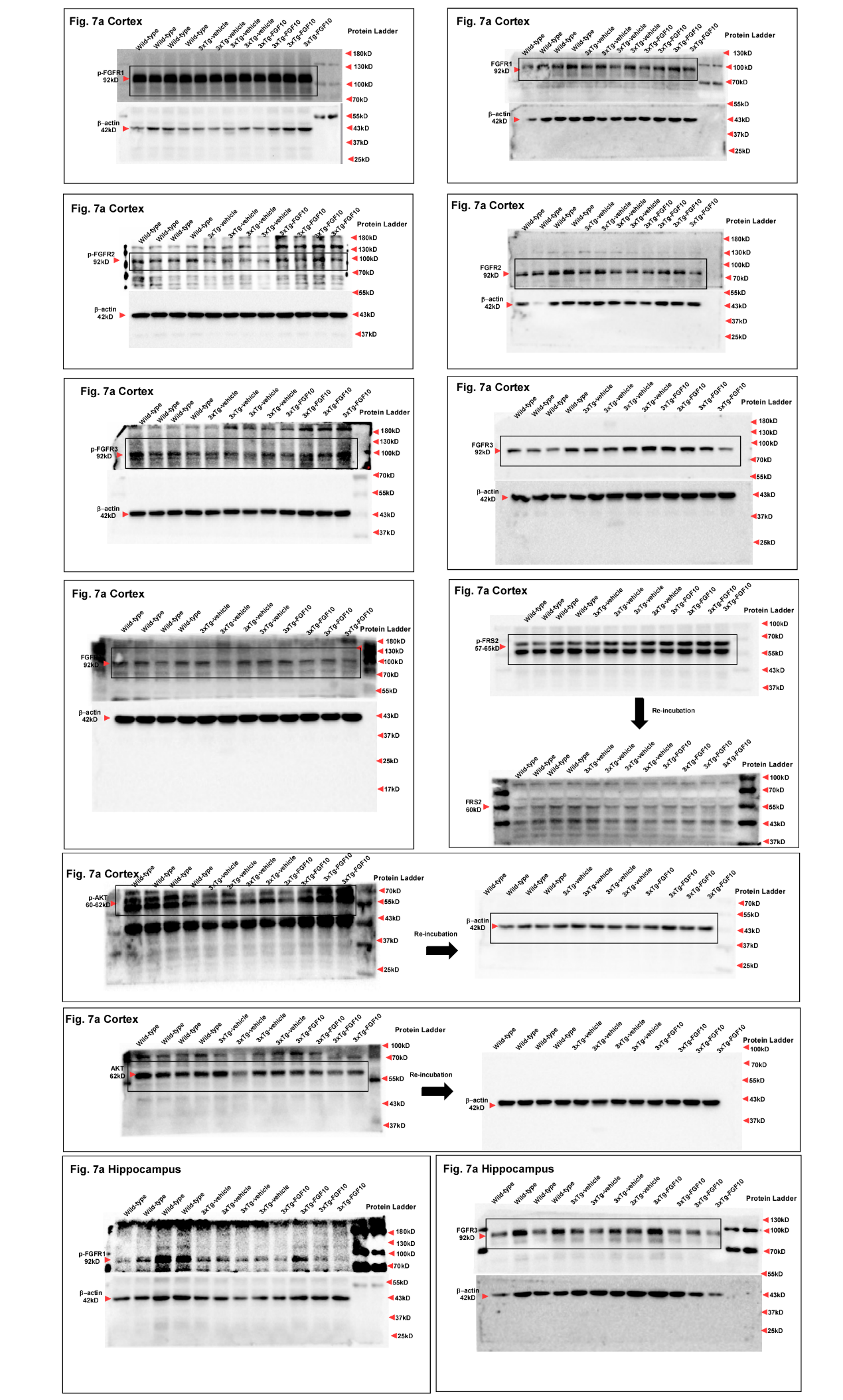


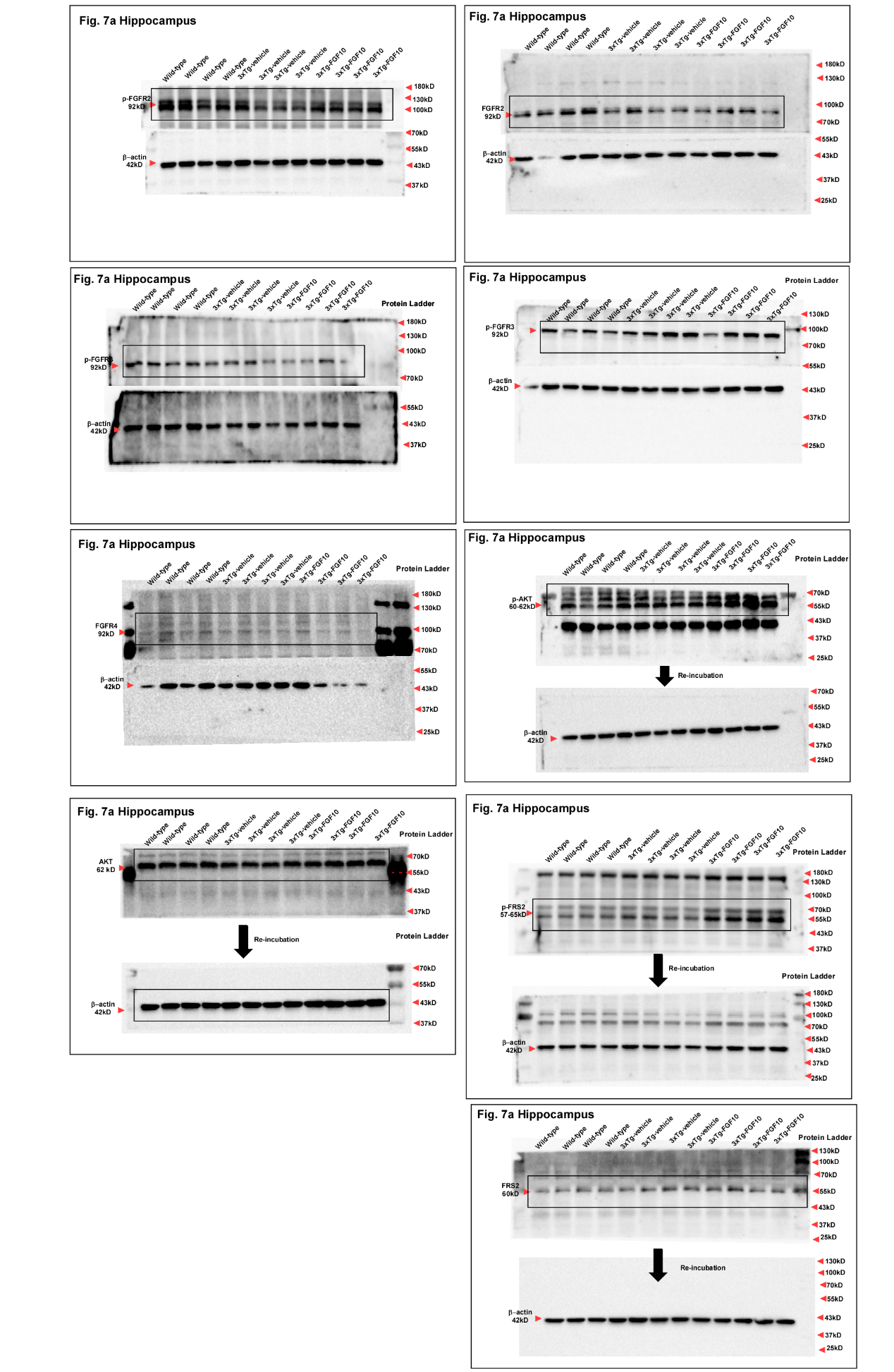


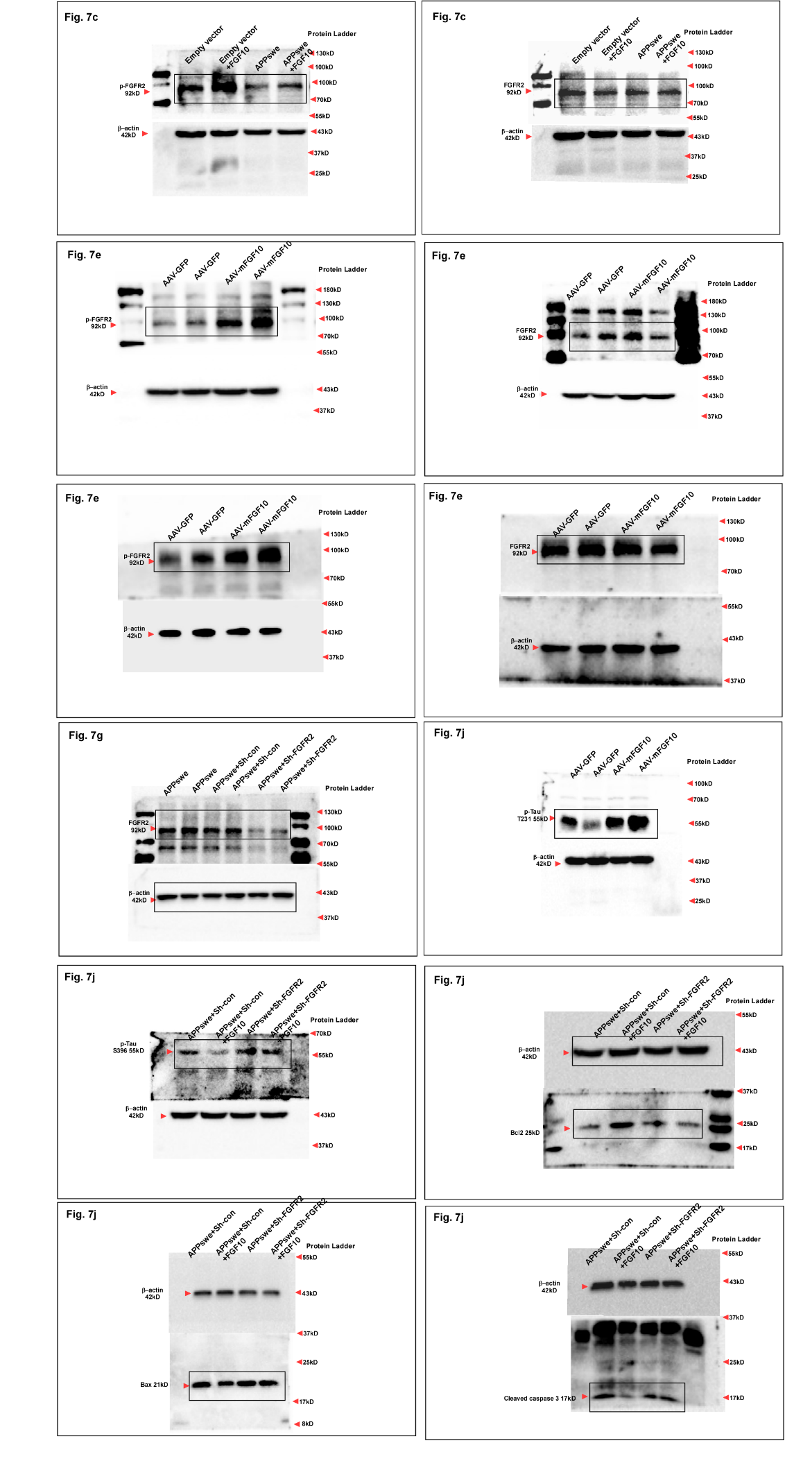


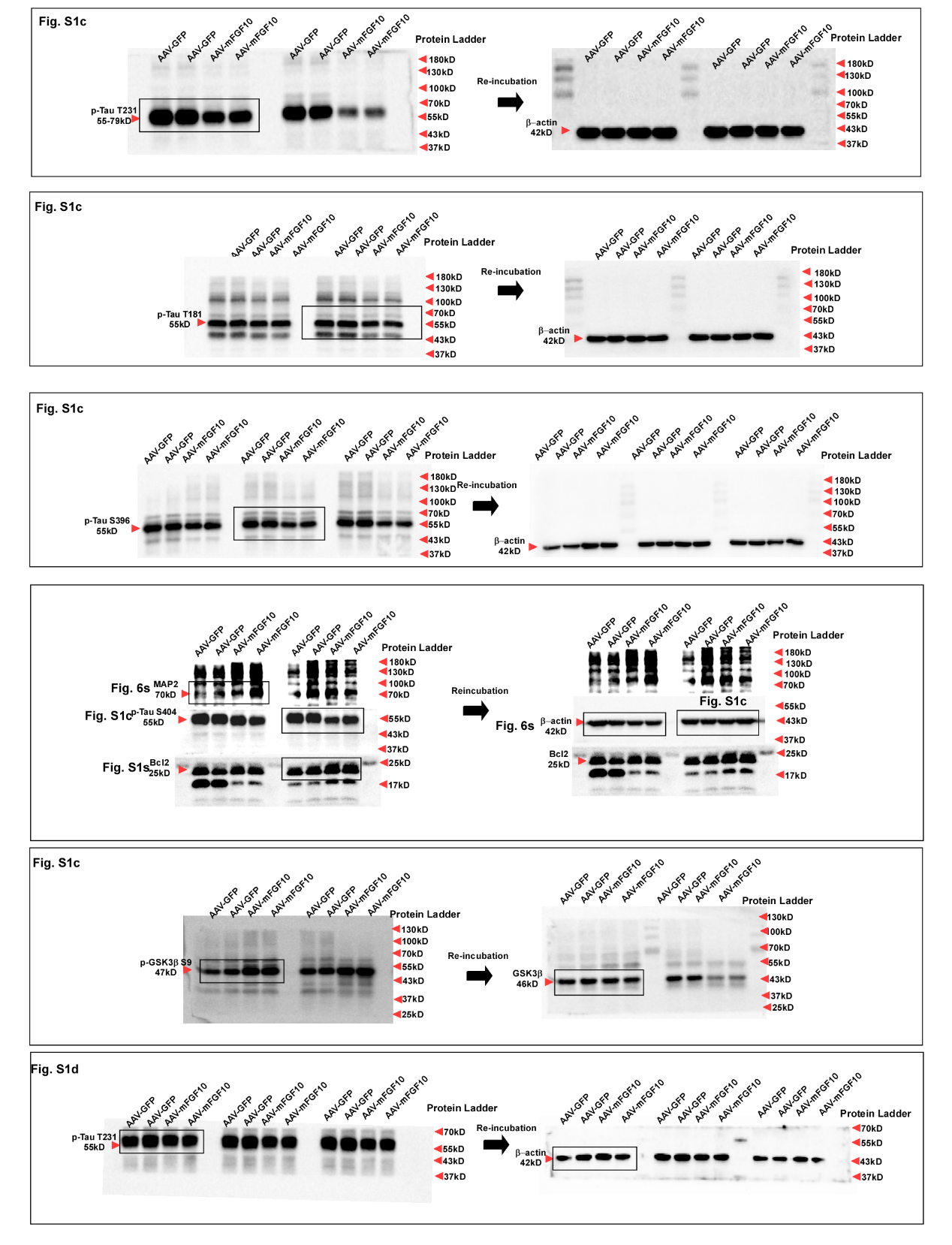

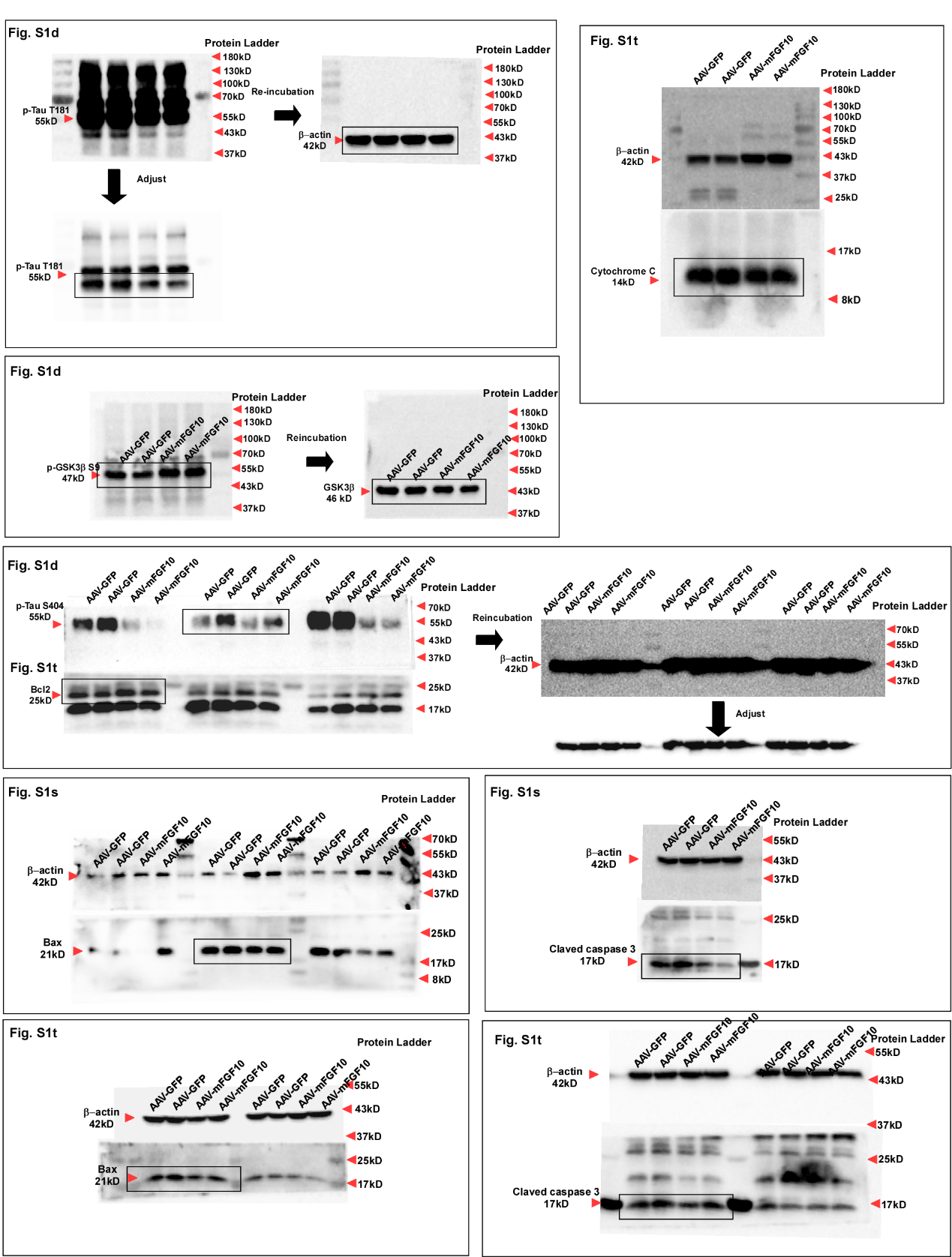


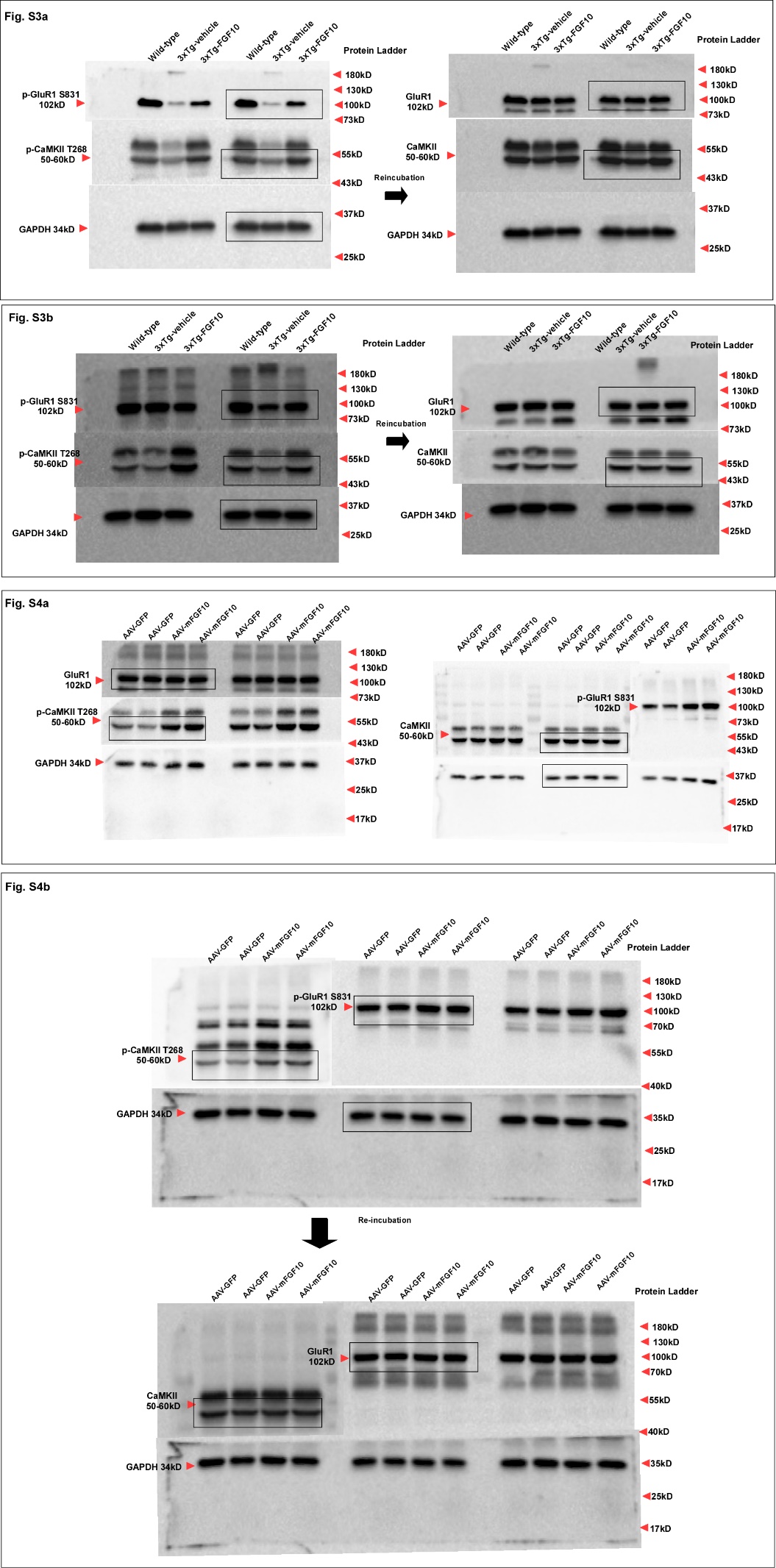

Supplement: Supplementary file 1 — Data S1. [file ACEL-22-e13937-s001.docx]
